# Alchemical Free Energy Methods Applied to Complexes of the First Bromodomain of BRD4

**DOI:** 10.1021/acs.jcim.1c01229

**Published:** 2022-03-08

**Authors:** Ellen E. Guest, Luis F. Cervantes, Stephen D. Pickett, Charles L. Brooks, Jonathan D. Hirst

**Affiliations:** † School of Chemistry, University of Nottingham, University Park, Nottingham NG7 2RD, U.K.; ‡ Department of Chemistry, University of Michigan, Ann Arbor, Michigan 48109, United States; ¶ Computational Chemistry, GlaxoSmithKline RD Pharmaceuticals, Stevenage SG1 2NY, U.K.

## Abstract

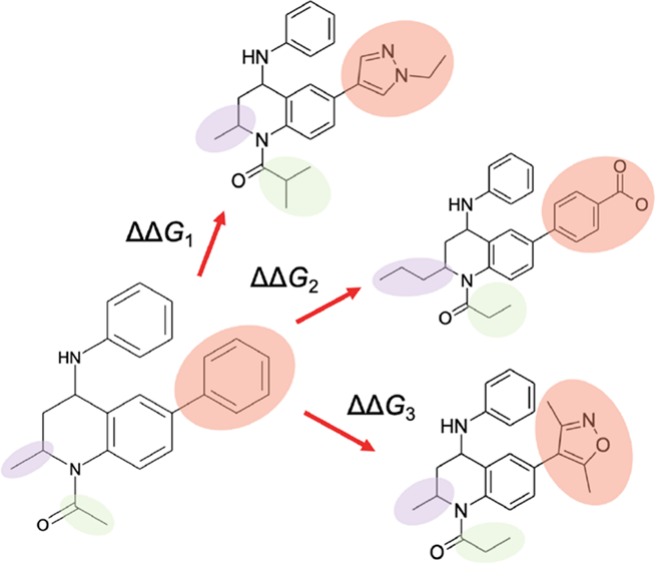

Accurate and rapid predictions of the binding affinity of a compound to a target are one of the ultimate goals of computer aided drug design. Alchemical approaches to free energy estimations follow the path from an initial state of the system to the final state through alchemical changes of the energy function during a molecular dynamics simulation. Herein, we explore the accuracy and efficiency of two such techniques: relative free energy perturbation (FEP) and multisite lambda dynamics (MSλD). These are applied to a series of inhibitors for the bromodomain-containing protein 4 (BRD4). We demonstrate a procedure for obtaining accurate relative binding free energies using MSλD when dealing with a change in the net charge of the ligand. This resulted in an impressive comparison with experiment, with an average difference of 0.4 ± 0.4 kcal mol^–1^. In a benchmarking study for the relative FEP calculations, we found that using 20 lambda windows with 0.5 ns of equilibration and 1 ns of data collection for each window gave the optimal compromise between accuracy and speed. Overall, relative FEP and MSλD predicted binding free energies with comparable accuracy, an average of 0.6 kcal mol^–1^ for each method. However, MSλD makes predictions for a larger molecular space over a much shorter time scale than relative FEP, with MSλD requiring a factor of 18 times less simulation time for the entire molecule space.

## Introduction

Alchemical free energy calculations are important in drug design and development.^1^ The accurate and reliable prediction of ligand binding free energies presents a way to minimize the number of compounds made in the laboratory, while also giving synthetic chemists the confidence to embark on novel and often challenging syntheses of molecules with the potential to be lead compounds. A common use of alchemical methods, such as free energy perturbation (FEP)^2,3^ and thermodynamic integration (TI),^4,5^ is in postdocking refinement, where more accurate predictions of binding affinity, compared to docking scores, are desired.^6,7^ This often involves small modifications made to a hit compound to increase its potency or improve physicochemical properties without compromising potency.

A reduction in the computational expense of alchemical methods would facilitate their use for the high throughput estimation of binding free energies in drug discovery projects, in both an industrial and academic setting. Lambda dynamics^8,9^ presents an opportunity to improve this throughput. These types of free energy calculations can predict the relative binding free energy (RBFE) for large sets of compounds in a small number of simulations. Multisite lambda dynamics (MSλD),^10^ an extension of lambda dynamics, also allows for modifications at multiple sites of a ligand scaffold in a single simulation, which is more realistic of the types of changes that are made to a compound in typical lead optimization projects.

Herein, the application of relative FEP and MSλD simulations to a set of molecules for the inhibition of the first bromodomain (BD1) of the bromodomain-containing protein 4 (BRD4) is explored. The accuracy of performing four MSλD calculations is assessed, compared to over 150 relative FEP calculations for the equivalent perturbations. This is considered in the context of the time saved in manual intervention required for the methods and their computational expense. In addition, some of the calculations involve a change in net charge. This is one of the most difficult aspects of alchemical calculations. We propose and validate a strategy for dealing with such changes.

BRD4 is a member of the bromodomain and extraterminal domain (BET) family. BET proteins play a crucial role in regulating gene expression.^11^ Furthermore, as histone acetylation readers, they contribute to tumorigenesis, making them important targets for the development of small molecule drugs to inhibit these epigenetic interactions. BRD4 is the most extensively studied member of the BET family, due to its promise as a therapeutic target for diseases such as cancer, neurodegenerative disorders, inflammation, and obesity.^12−19^ As a result, the computational chemistry guided synthesis of new compounds is being used (in various guises) by several teams. In the first study on absolute binding free energies for a diverse set of drug-like molecules, Aldeghi et al.^20^ studied a set of BRD4 inhibitors using absolute FEP simulations. Although this method was considered, at the time of the study, too computationally intensive to be feasibly integrated into lead optimization projects, the highly accurate binding predictions, a mean absolute error of 0.6 kcal mol^–1^, demonstrated the amenability of BRD4 to alchemical calculations.

**Figure 1:**
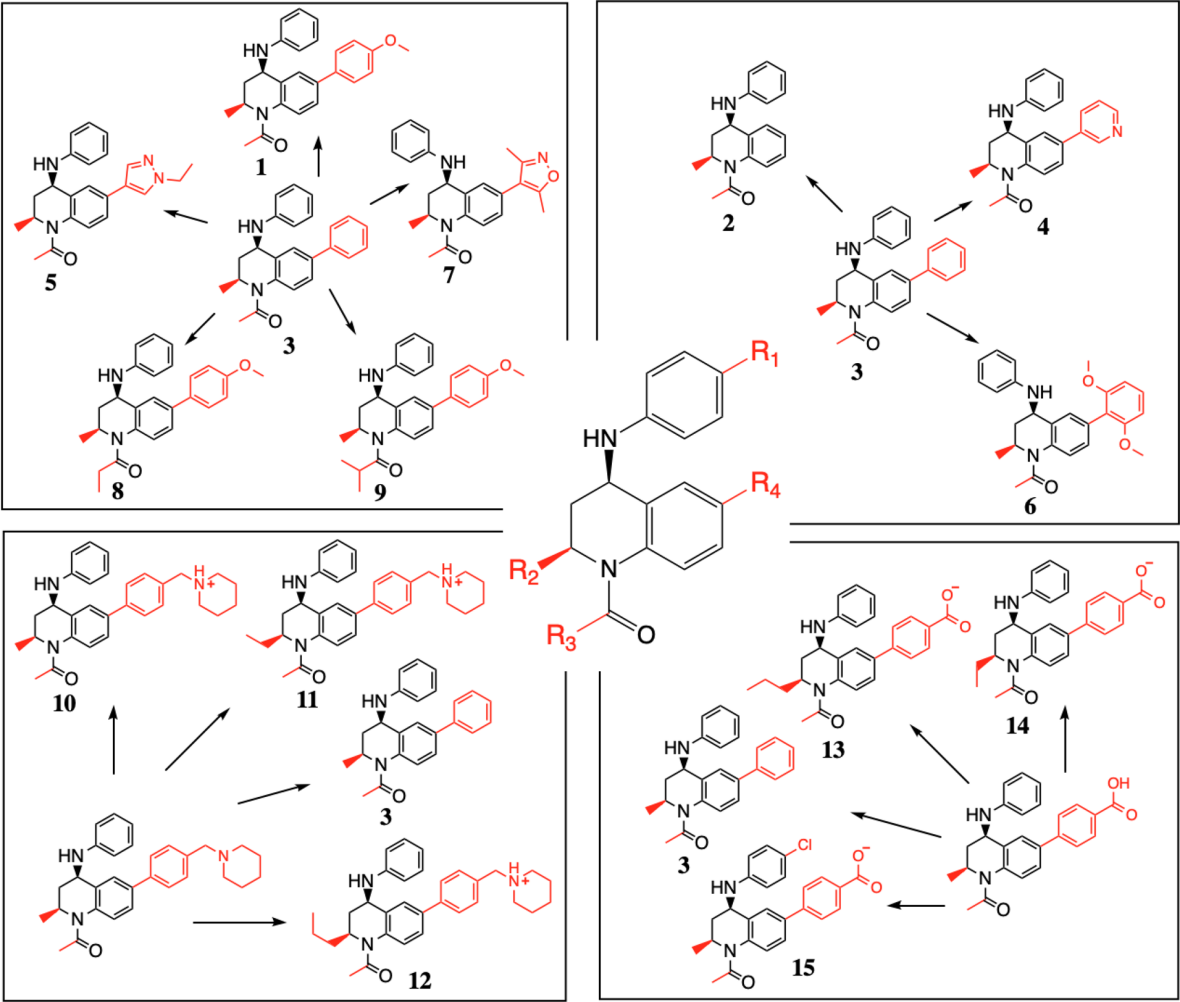
Compound in the center shows the THQ scaffold of a series of BRD4-BD1 inhibitors. In the four boxes are the groups of perturbations performed using MSλD.

For the assessment of relative FEP and MSλD approaches to this system, we use a set of inhibitors that has been previously studied *in silico* by Coveney and co-workers.^21^ The compounds studied (Figure 1) are based on a tetrahydroquinoline (THQ) scaffold and represent a good range of chemical functionality and binding affinities. There are four points of substitution, which we refer to as sites 1 to 4. All derivatives of the scaffold have a net neutral charge except for those with the benzoic acid and piperidine substituents at site 4. These groups are charged under physiological conditions and present an opportunity for the refinement of RBFE calculations that involve a change in charge.

Experimental binding affinities are available for 15 THQ compounds, based on different combinations of the substituents on the scaffold. These have a pIC_50_ range of ≤4.3 to 7.9, which corresponds to a binding free energy range of ∼5 kcal mol^–1^.^21^ This range in activity, coupled with the relatively small modifications on each of the sites, makes this series of compounds a good test case for RBFE calculations. Wan et al.^21^ described binding free energy calculations on this series using two free energy protocols. The first approach was termed “enhanced sampling of molecular dynamics with approximation of continuum solvent” (ESMACS)^22^ and is based on MM-PBSA, where the solvent is treated implicitly. The second approach involved TI with enhanced sampling (TIES).^23^ ESMACS was used for the full set of compounds, while the TIES calculations were split into three subsets of compounds, so that perturbations involved derivatives with the same net charge. A good correlation with experimental data was found, with a Spearman rank correlation coefficient, *r*_*s*_, of 0.78 for the ESMACS 3-trajectory calculations and 0.92 for TIES. Furthermore, the ESMACS protocol showed good reproducibility, with a Spearman correlation of 0.98 ± 0.02 between two independent studies performed on different supercomputers.

**Figure 2:**
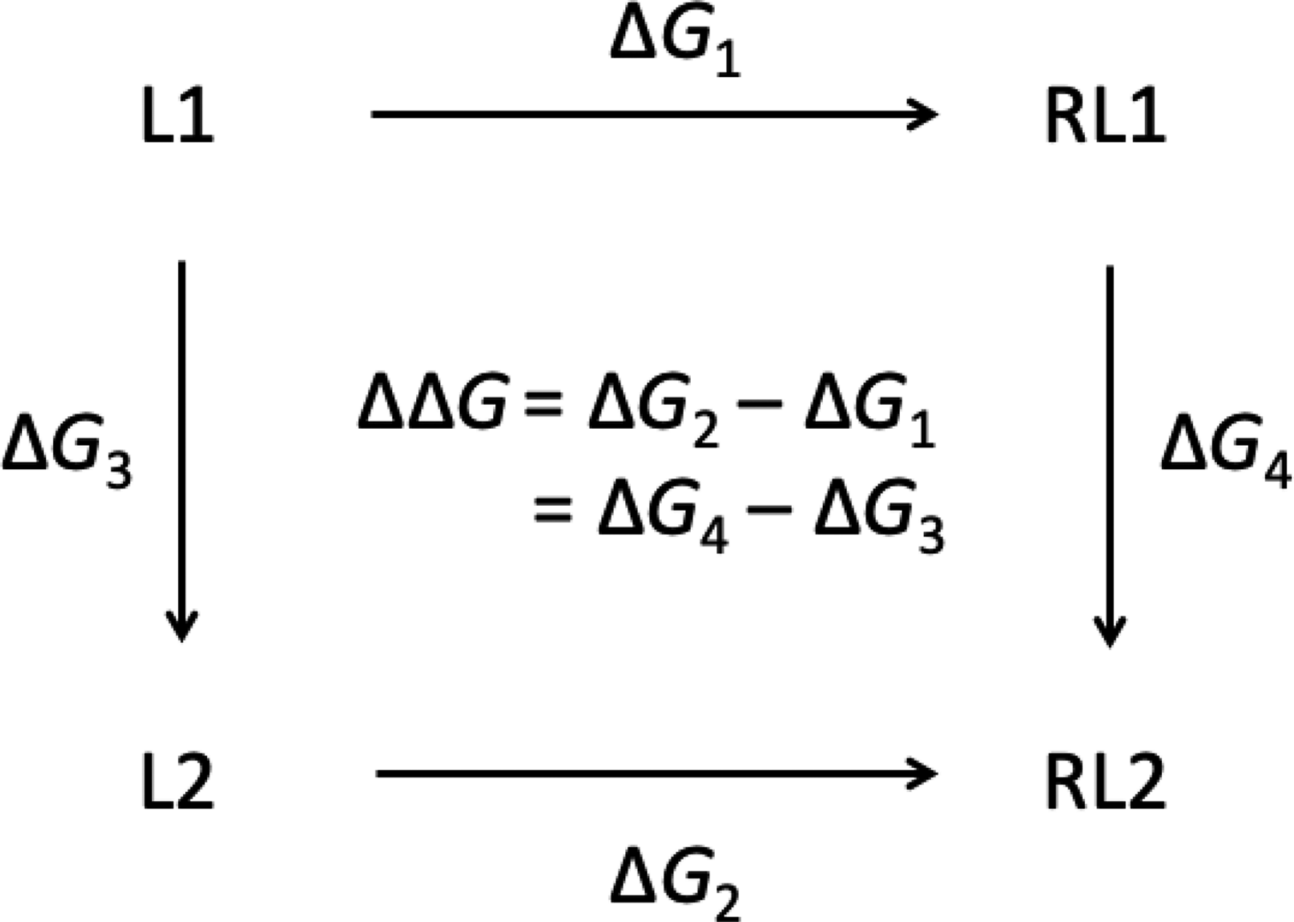
A thermodynamic cycle describing the binding of two ligands, L1 and L2, to a receptor, R. The relative free energy of binding can be calculated from either the physical (Δ*G*_2_ – Δ*G*_1_) or alchemical (Δ*G*_4_ – Δ*G*_3_) legs of the cycle. In FEP calculations, the transformations of the alchemical pathways are modeled.

In this study, we investigate how the calculation of RBFE compares when using relative FEP^2,3^ and MSλD^10^ protocols. Relative free energy calculations involve constructing a thermodynamic cycle so that the vertical legs involve making a simple modification to a ligand, with the compound in the solvent phase on one side and the compound in complex with the receptor on the opposite side of the cycle (Figure 2). The change in free energy for each of these alchemical transformations is measured. Providing that the overall binding mode of the ligand is conserved, it is possible to determine the relative difference in the free energy of binding, ΔΔ*G*, between the two derivatives. FEP simulations use a series of alchemical intermediate states, called λ windows, to calculate the free energy change for each vertical leg of the thermodynamic cycle. Force field parameters assigned to the “disappearing” atoms on the ligand are slowly decoupled from the system, while parameters for the “appearing” atoms are introduced, with the progression of the λ windows. Within this study, the optimal number of λ windows and simulation time is assessed for this BRD4-BD1 system.

In contrast to FEP, TI or slow-growth, nonequilibrium methods,^24^ MSλD calculations utilize λ as a dynamic variable that propagates throughout a simulation, along with the coordinates.^25^ Similar to relative FEP calculations, lambda dynamics is particularly applicable when applied to lead optimization tasks where knowing the difference in binding affinity between small changes on a common scaffold is required. However, by introducing additional λ coordinates, one can estimate the relative ΔΔ*G* values of multiple different variations of a scaffold in a single simulation, obviating the need to do a separate simulation for each pairwise set of compounds. Furthermore, in MSλD, it is possible to perform perturbations simultaneously on more than one substitution site of a scaffold, and ΔΔ*G* values for the combinatorial set of substituents are obtained, with the consequence that MSλD simulations can be significantly more efficient than traditional FEP calculations.^26^ This concept is demonstrated herein, where the computational expense and accuracy of these methods are investigated.

## Materials and Methods

### Molecular Docking

Receptor coordinates were taken from the X-ray crystal structure (PDB: 4BJX) of BRD4-BD1 in complex with a small molecule inhibitor, I-BET726,^21^ which is the compound in this series with the best binding affinity. Prior to docking, the protein structure was minimized for 20,000 steps using a conjugate gradient and line search algorithm and equilibrated for 1.5 ns in the NVT ensemble and 18.5 ns in the NPT ensemble. The cocrystallized ligand was retained for the equilibration period. Solvation and periodic image setup for the equilibration period is outlined in the relative FEP methodology section below. Once the protein structure was equilibrated, all water molecules were removed, with the exception of the highly conserved network of five water molecules, which line the binding pocket of BRD4-BD1. These water molecules are important for ligand binding and stabilizing the protein structure.^27−30^ Furthermore, previous work using molecular docking and absolute free energy perturbation showed good agreement with reproducing experimental binding poses and binding free energies when retaining this water network and removing any remaining crystallographic water molecules.^31^ Using receptor generation software from the OpenEye docking toolkit,^32,33^ I-BET726 was assigned as the ligand and is treated as noninteracting during the molecular docking. A box centered around the original ligand with sides of length 17.7 × 19.7 × 17.0 Å was situated to cover the BRD4-BD1 binding cavity fully, giving a total receptor volume of 5906 Å^3^. The 15 THQ compounds were protonated according to physiological pH and prepared using OpenEye OMEGA.^33^ Conformers were generated using a truncated form of the MMFF94s force field^34^ with a maximum energy difference of 20 kcal mol^–1^ set from the lowest energy conformer. A maximum of 1000 conformers was allowed, and those within 0.5 Å RMSD of any others were considered duplicates and removed. Docking was performed using OpenEye FRED^32^ using the high resolution setting with rotational and translational step sizes of 1 Å. Once docked, OpenEye FRED provides ten sets of ligand coordinates that display the best docking scores. With the exception of compound **7**, all compounds exhibited one conserved binding mode, with little variation between each set of the ten best coordinates. Within the small movements of this binding mode, the pose taken forward for each compound was chosen to optimize the overlap between the common core of the THQ scaffold. Compound **7** displayed two binding modes, with the common binding pose also taken forward for free energy of binding evaluation.

### Multisite λ-Dynamics Simulations

**Figure 3:**
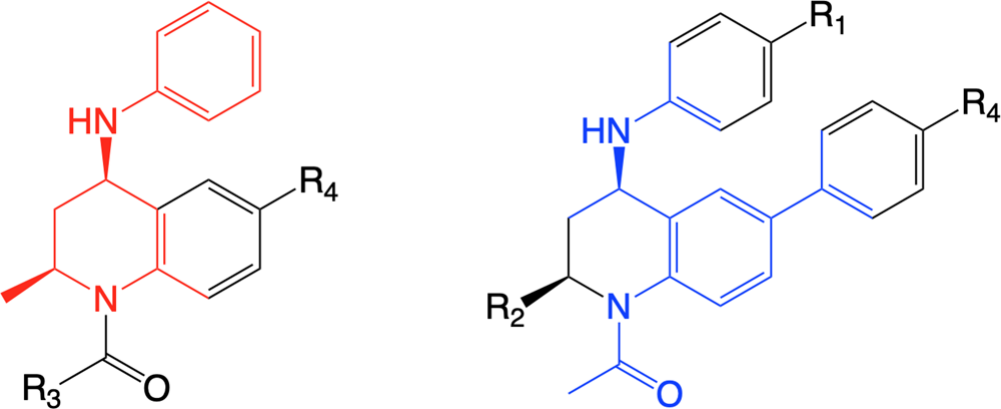
Common cores used in MSλD calculations. Atoms in red were used as the core for calculations involving neutral substituents. Atoms in blue were used as the core for charged substituents. All other atoms on the THQ scaffold are treated as substituents or anchor atoms, which are perturbed during MSλD.

Atoms belonging to all derivatives of the THQ scaffold were identified using a maximum common substructure (MCS) search. The common core used for the neutral set of substituents is shown in red in Figure 3, and the core used for the charged substituents is shown in blue. All remaining atoms were fragments or anchor atoms, which are coupled and decoupled from the system as their corresponding λ variables propagate through the simulation. Fragments correspond to the parts of the compound that are treated as substituents. Anchor atoms are the attachment points between the common core and the fragments and become part of the substituents once the simulation is initiated. Once an initial common core was identified, the core, fragments, and anchor atoms were manually altered so that additional atoms became part of the fragments. Although all atom types on the amide and THQ groups of the ligand scaffold are consistent, regardless of the substituents, not all atoms are chosen to belong to the common core. This is to allow a change in the partial charges assigned to each of the atoms, which are affected by the substituent attached, thereby enabling a better representation of the electrostatics of the ligand.

During λ dynamics, the charge of the compound must sum to an integer net charge, regardless of the combination of substituents at each site. Therefore, the partial charges of substituents at one particular site are normalized so that each substituent has the same total net partial charge. An exception is when a charge perturbation is performed, with the addition of a protonated or deprotonated substituent. The alteration of partial charges for the preparation of λ dynamics is termed charge renormalization and is performed using an algorithm developed by the Brooks group (Supporting Information). Initial partial charges were obtained from atom type matching with existing parameters in the CHARMM force field, using CGenFF.^35^ There was an average RMSD of 0.015 *e* between the original CGenFF charges and the adjusted charges. All other parameters, attributed to bond lengths and angles and dihedral angles, remained unchanged from the CGenFF initial parameter assignments.

Two systems were built, one composed of the ligand in solution and the second with the ligand in complex with BRD4-BD1 (PDB: 4BJX(21)). The ligand, receptor, and solvent coordinates for the complex site were obtained from the equilibrated structure and molecular docking, as detailed above. Ligand topologies were constructed using a multiple topology approach.^8,36^ This is a similar method to the dual topology approach in FEP, where all substituents explicitly exist in the topology, attached to the same common core. The hybrid multitopology models used in MSλD are created without internal energy terms that span two substituents. This is achieved through the delete connectivity command in CHARMM.^37^ For the ligand in solvent system, the ligand was solvated in a cubic periodic boundary cell with 1755 TIP3P water molecules.^38^ All simulations were performed using the CHARMM molecular simulation package with the domain decomposition (DOMDEC) computational kernels on GPU.^37,39,40^ MD simulations were run in the NPT ensemble at 298 K and 1 atm using a Nosé–Hoover thermostat^41^ and Langevin pressure piston with a friction coefficient of 20 ps^–1^.^42^ A time step of 2 fs was used, with hydrogen-heavy atom bond lengths constrained with the SHAKE algorithm.^43^ A cutoff distance of 12 Å was used for van der Waals pairs, with a switching function at a distance of 10 Å. The electrostatic potential energy was computed using the Particle Mesh Ewald method.

The THQ compounds were split into four sets as shown in Figure 1: compounds with a net neutral charge were split into two groups, those with a net charge of +1 (compounds **10**–**12**) and finally those with a net charge of −1 (compounds **13**–**15**). Considering only compounds with a net neutral charge, there is one substituent at site 1, one at site 2, three at site 3, and seven at site 4. Similar to FEP, the accuracy of MSλD is impacted by the sizes of the perturbations. Although there are no hard rules about the number of substituents or sites that can be handled, generally, the smaller the perturbation between each substituent, the more substituents or sites that can be used. In our data set, seven quite varied substituents on one site, along with other sites of substitution, means that it is sensible to split it into two sets of calculations. Substituents on site 4 were split into two groups based on their similarity, with the phenyl, methoxyphenyl, isoxazole, and ethylpyrazole substituents in one group and the phenyl, hydrogen, pyridyl, and dimethoxyphenyl substituents in the second. The phenyl substituent was included in both sets as the reference compound. For comparison, a single MSλD calculation with all neutral substituents was performed. Similar ideas have been utilized in a recent large-scale benchmarking of MSλD by Raman et al.^44^

For all MSλD calculations, adaptive landscape flattening^26,36^ (ALF) was used to enhance the sampling. To estimate differences in free energies accurately, it is necessary to have sufficient sampling of all physically meaningful end states. In alchemical transformations, sampling can be limited by high energy barriers, and so ALF is applied to calculate the biases needed to flatten the energy surface between end points. A soft-core potential was also used to scale all nonbonded interactions by λ and to prevent end-point singularities.^36,45,46^ To identify initial biases for the complex system, 50 serial simulations of 100 ps each were performed, followed by 30 simulations of 1 ns to refine the biases. ALF was performed for the ligand in solution for 50 simulations of 100 ps, followed by 20 simulations of 1 ns. Production simulations were run for 20 and 50 ns for the solution and complex systems, respectively, with the first 5 ns of each discarded as equilibration. Five replicas of each production run were performed using a different random seed. End-state populations were binned using a λ ≥ 0.99 cutoff criterion, and the final relative free energy of binding values were calculated by Boltzmann reweighting end-state populations to the original biases and then using eq 1.^8,9^ Uncertainties were calculated as the standard deviation of the mean value over the five independent runs.
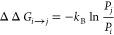
1

Eq 1 shows how relative binding free energies are calculated as the ratio of the amount of time one ligand is sampled compared to a reference ligand. In our calculations, compound **3** was chosen as the reference ligand, because the hydrogen atom at site 1 and methyl groups at sites 2 and 3 are the most common substituents at these sites across all of the compounds. Furthermore, the phenyl group at site 4 is most similar to all other substituents at this position and therefore involves the smallest perturbation between substituents. To check that there had been sufficient sampling of unsymmetrical substituents at site 4, the dihedral angle around the site 4-phenyl bond was measured along the trajectory. These figures can be found in the Supporting Information.

As changing the net charge of the compound adds a layer of complexity, MSλD calculations involving charged substituents were constructed in a different way to the neutral substituent calculations. Separate simulations were performed with the neutral form of each charged substituent as the reference compound. For example, for the negatively charged compounds, benzoic acid was used as the reference substituent on site 4. The deprotonated form, benzoate, was included as a substituent for MSλD. Substituents attributed to compound **3** were also included in the MSλD calculation, so that the relative binding free energy with respect to compound **3** could be calculated, for consistency. Using benzoic acid on site 4 as the reference, compared to a phenyl group, meant there was a smaller perturbation and the change in net charge could be accounted for more effectively. The same approach was used for compounds with a piperidine substituent, which is protonated at physiological pH.

### Relative Free Energy Perturbation Simulations

**Figure 4:**
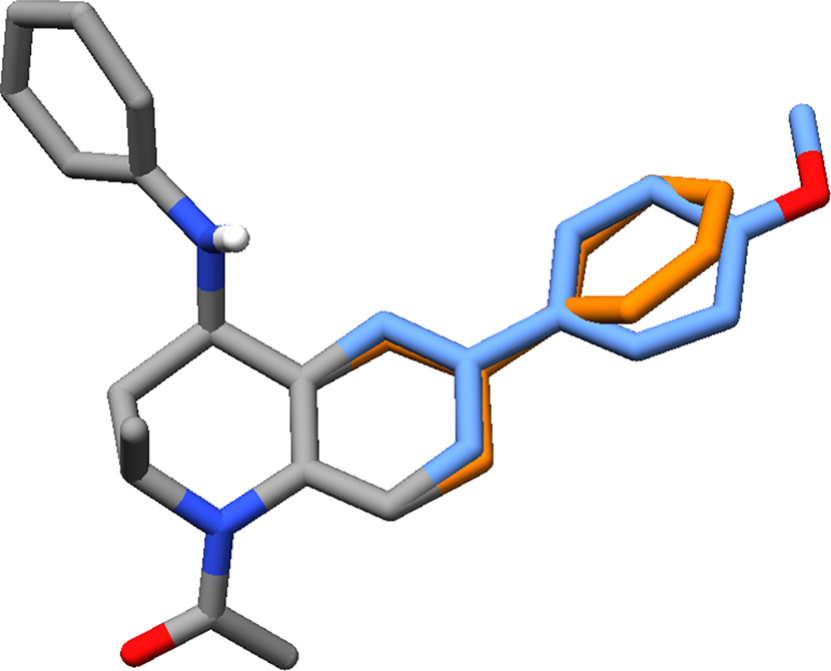
Dual topology constructed for the alchemical transformation of a phenyl group (orange) to a methoxybenzene group (blue), attached to a THQ scaffold (gray).

Dual topologies were constructed, with compound **3** as the reference compound, for each alchemical transformation. For example, Figure 4 shows the ligand topology for the transformation of compound **3** to compound **1**. When λ = 0, the phenyl group is interacting with the system, and when λ = 1, the methoxybenzene is interacting. Using input generated by CHARMM-GUI,^47^ all complex systems were solvated in a cubic periodic boundary cell with edge distances of 18 Å to construct an explicitly modeled solvent consisting of around 22,000 TIP3P water molecules.^38^ Depending on the net charge of the ligand, Na^+^ or Cl^–^ ions were added, to neutralize the system. To optimize the solvent positions, all heavy atoms were fixed, except for water molecules, during 50 steps of steepest descent and 50 steps of Adopted Basis Newton–Raphson minimization. Potential energy evaluations were performed with the CHARMM force field.^48^ To ensure a fair comparison of binding free energies obtained from FEP and MSλD calculations, the charge renormalized ligand parameters, adapted from CGenFF,^35^ were used. Systems containing the ligand in solution, without the receptor, were also set up using input from CHARMM-GUI.^47^ Ligands were solvated in a cubic periodic boundary cell with around 2,300 TIP3P water molecules. Minimization and equilibration were performed using the same protocol as for the protein–ligand complexes.

Once set up, all systems were minimized for 20 ps using a conjugate gradient and line search algorithm using the NAMD simulation software.^50^ Protein backbone and side chain restraints were applied using harmonic constraints with force constants of 10 kcal mol^–1^ Å^–2^ and 5 kcal mol^–1^ Å^–2^ during a heating period of 50 ps. Systems were heated to 298 K in increments of 10 K. Restraints were removed for 0.1 ns of equilibration in the NVT ensemble and 4.9 ns in the NPT ensemble, with a 2 fs time step. The temperature was controlled using Langevin dynamics parameters, with a friction coefficient of 5 ps^–1^ for all equilibration and FEP simulations. Constant pressure was maintained using the Langevin piston Nosé–Hoover method^42^ with a target pressure of 1 atm. During equilibration, a cutoff distance of 12 Å was used for van der Waals pairs, with a switching function at a distance of 10 Å. Long-range electrostatic interactions were computed using the PME method.^51^ The SHAKE algorithm^43^ was also used.

To develop an efficient protocol for FEP calculations on these BRD4 inhibitors, a series of benchmark calculations were performed. The relative free energy of binding of compound **1**, with respect to compound **3**, was calculated using 8, 10, 16, 20, and 25 λ windows. For each λ window, 2 ns of equilibration was performed, followed by 1 ns of data collection. Electrostatic interactions of outgoing atoms were decoupled from the system from λ = 0 to λ = 0.5, while the electrostatics for incoming atoms were coupled to the system from λ = 0.5 to λ = 1. For all simulations, a soft-core potential was used to avoid “end-point catastrophes”. The effect of reducing the length of the data collection period for each λ window was then tested by performing the perturbation with 20 λ windows, 2 ns of equilibration, and 0.5 ns of data collection. Finally, equilibration of lengths 1 and 0.5 ns were tested, using 20 λ windows and 1 ns of data collection. The average value over three replicas was calculated for each combination of FEP parameters, with free energy values evaluated using the BAR method^52^ as implemented in the ParseFEP tool in VMD.^53^

**Figure 5:**
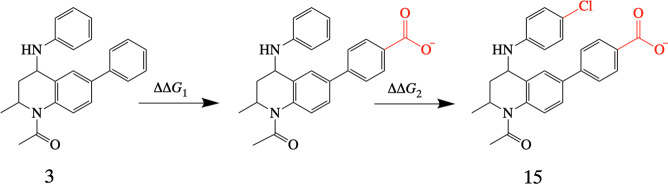
To calculate the binding free energy of compound **15**, relative to compound **3**, an intermediate step is required. The relative binding free energy is the sum of ΔΔ*G*_1_ and ΔΔ*G*_2_. Substituents being added or transformed are shown in red.

Once the optimal number of λ windows, equilibration length, and data collection length were established, the relative free energies of binding were calculated for the remaining compounds. As substituents on two sites of the common scaffold are modified, compared to compound **3**, for compounds **8**, **9**, and **11** to **15**, an intermediate FEP step was required. For example, to calculate the relative free energy of binding of compound **15**, FEP calculations were performed for the changes shown in Figure 5. First, site 4 was perturbed from a phenyl group to a benzoic acid substituent. In a separate simulation, the hydrogen atom on site 1 was then transformed to a chlorine substituent. The sum of the free energy changes for these transformations resulted in the total relative free energy of binding of compound **15**, with respect to compound **3**. Compound **1** served as the reference for transformations to compounds **8** and **9**, and compound **10** was the reference for transformations to compounds **11** and **12**. Therefore, including replicas, reverse transformations, and ligand in solution simulations, to obtain the full RBFE data set for the 14 compounds, with respect to compound **3**, a total of 168 FEP simulations were required.

## Results and Discussion

Relative FEP parameters such as number of λ windows, equilibration, and data collection length are often a balance between obtaining sufficient sampling of each λ state, while keeping the calculation to a reasonable time scale. Therefore, we first present our findings for the most effective parameters to use for our system of interest. Next, we discuss the calculation of the biasing potentials for the MSλD calculations. On demonstration of the reliability of our procedures, we compare the accuracy of relative FEP and MSλD with respect to experimental binding affinities. Lastly, an assessment of the investment required for each method, in terms of both computational and human time, is presented.

### Relative FEP Benchmarking

To establish the best number of λ windows to use for relative FEP calculations on this series of BRD4 inhibitors, perturbations from compound **3** to compound **1** were performed with 25, 20, 16 10 and 8 windows. This alchemical perturbation involved the transformation of a phenyl substituent on site 4 of the THQ compound to a methoxybenzene substituent. To assess the performance of the calculations, three criteria were taken into account. First, a comparison between the predicted relative free energy of binding and the experimental value was made. Second, the standard deviation of the mean BAR free energy estimate over three independent replica runs was calculated. Third, the convergence was measured by plotting the relative binding free energy calculated using an increasing fraction of the simulation data. The free energies using the reverse proportion of the data were also plotted. Convergence plots are important for ensuring that the free energy is being measured for an equilibrated system. This graphical method of assessing convergence, outlined by Klimovich et al.,^54^ helps identify any nonequilibrated regions throughout the simulation.

**Figure 6:**
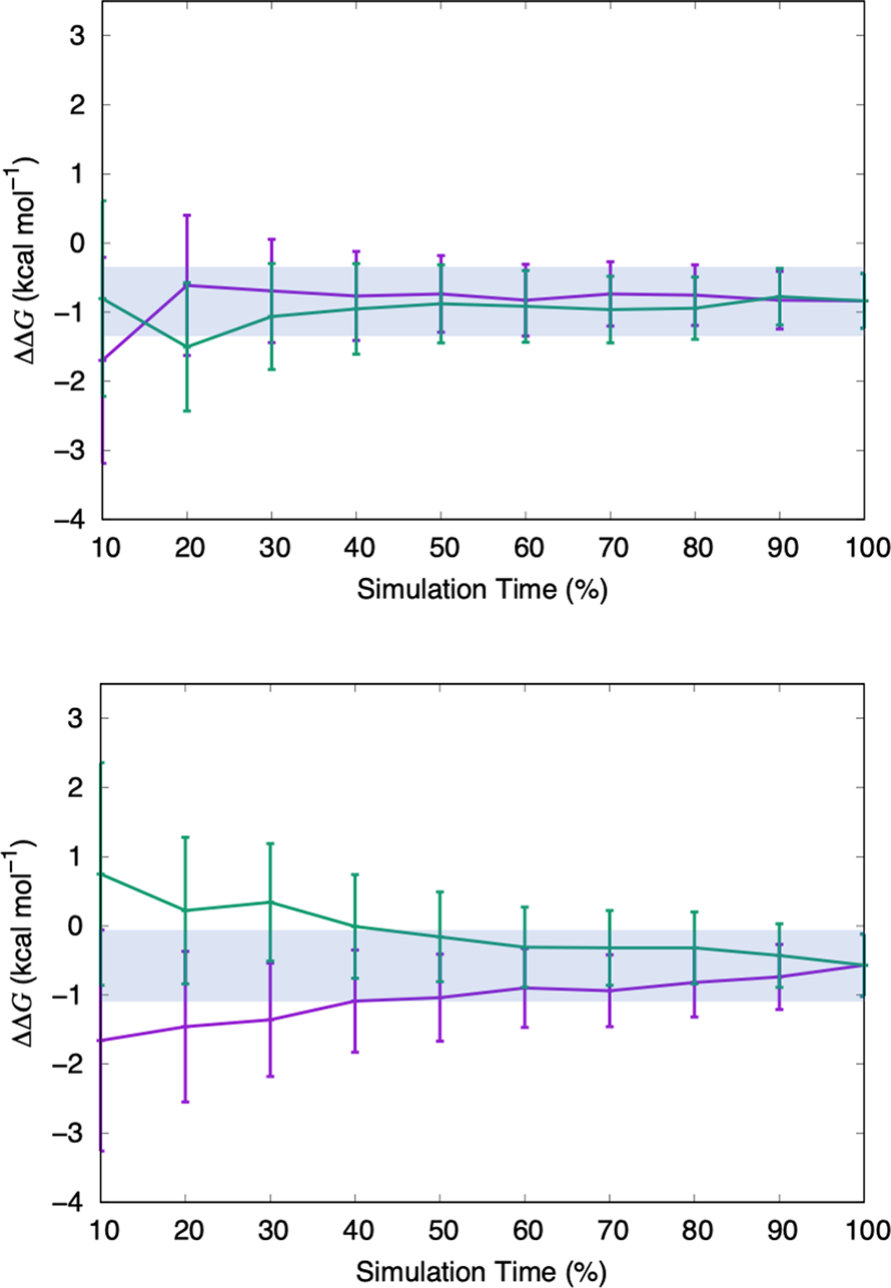
Convergence assessment of the transformation of a phenyl substituent at site 4 to a methoxybenzene substituent. (Top) Using 20 λ windows with 2 ns of equilibration and 1 ns of data collection. (Bottom) Using 16 λ windows with 2 ns of equilibration and 1 ns of data collection. The forward (purple line) and the reverse (green line) simulation time series are shown. The horizontal shaded bar indicates the equilibrated region.

Table 1 shows the mean predicted relative binding free energies over three replicas, their errors, and the absolute difference with experimental values. All predicted values are within chemical accuracy of the experimental values, which is generally considered to be 1 kcal mol^–1^. However, there is an increase in their absolute differences with a decreasing number of λ windows. Furthermore, the error also increases. This is to be expected, as decreasing the number of intermediate steps between the transformation means that there will be a poorer overlap of phase space between each window. For reliable estimations, an error of no more than 0.5 kcal mol^–1^ is desirable. This corresponds to a variation in a pIC_50_ value of approximately 0.4. Although FEP with 25 lambda windows results in the lowest error of 0.3 kcal mol^–1^, using 16 or 20 windows still gives acceptable errors of ≤0.5 kcal mol^–1^. Additionally, using fewer windows results in a saving of computational time. With this in mind, FEP with 16 or 20 λ windows appears to be the best approach. Figure 6 shows the convergence plots for these perturbations. Convergence plots for all benchmark FEP calculations can be found in the Supporting Information. An agreement, within error, between the forward and reverse free energies is a sign of an equilibrated system. The shaded bar on the plots indicates an error range of 0.5 kcal mol^–1^, centered on the final relative free energy value. These plots show that FEP with 20 λ windows results in free energies that are better converged. Therefore, relative binding free energies in this study are predicted using 20 intermediate steps between the initial and final states.

**Table 1:** Benchmarking of Relative FEP Protocols^a^

λ windows	equilibration (ns)	data collection (ns)	ΔΔ*G*_calc_ (kcal mol^–1^)	error (kcal mol^–1^)	absolute difference (kcal mol^–1^)
25	2	1	–0.5	0.3	0.2
20	2	1	–0.8	0.4	0.5
16	2	1	–0.6	0.5	0.3
10	2	1	–1.2	0.6	0.9
8	2	1	0.3	0.6	0.8
20	2	0.5	–0.8	0.6	0.5
20	1	1	–1.1	0.4	0.8
20	0.5	1	–0.5	0.4	0.2

a Varying numbers of λ windows, equilibration time, and data collection time were tested. RBFE predictions are compared to experiment. Errors are calculated as the standard deviation of free energy estimates over three replicates.

In an attempt to gain computational speed, perturbations with data collection periods of 0.5 ns for each λ window were tested. This resulted in an error of 0.6 kcal mol^–1^ (Table 1). Furthermore, poor convergence was observed. Therefore, 1 ns of data collection for each λ window was performed for all FEP calculations. Equilibration periods of 1 and 0.5 ns were also tested for each λ window. Reducing the equilibration of the windows to 0.5 ns did not affect the error or convergence of the predicted relative binding free energies. Therefore, we conclude that a protocol of using 20 λ windows with 0.5 ns of equilibration and 1 ns of data collection results in a good compromise between accuracy and computational efficiency.

### Adaptive Landscape Flattening

**Figure 7:**
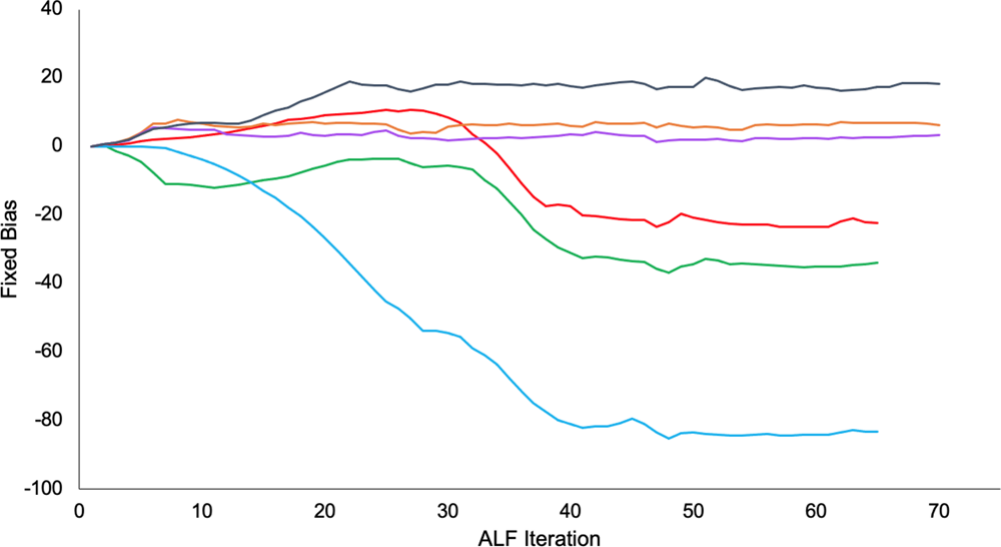
Convergence of the fixed bias for each substituent at site 4 as the ALF simulations progress. Substituents at site 4 include methoxyphenyl (red), ethylpyrazole (green), isoxazole (light blue), hydrogen (orange), pyridyl (purple), and dimethoxyphenyl (dark blue).

ALF is the process of calculating the biases to flatten the alchemical potential energy landscape between substituents on a given site, to ensure sufficient sampling of all substituents.^26,36^ To assess the fixed biases that were used for MSλD, their convergence along the serial ALF simulations was investigated. Figure 7 shows that at the end of each ALF process, the biases were stable and therefore suitable to be used for data collection.

### Relative Binding Free Energies

#### Accuracy and Reliability

Relative binding free energies are shown in Table 2. Results shown for the neutral compounds using MSλD are RBFEs calculated from splitting the compounds into two separate calculations, as this improved the accuracy. RBFE predictions when including all substituents in one calculation can be found in the Supporting Information. Overall, the two methods have similar levels of accuracy compared to experiment. MSλD calculations resulted in an average difference of 0.6 ± 0.7 kcal mol^–1^ to experiment, and for relative FEP predictions this was 1.0 ± 1.3 kcal mol^–1^. Furthermore, when discounting the large deviation from experiment found for compound **9**, the average differences for the MSλD and relative FEP calculations become 0.6 ± 0.7 kcal mol ^–1^ and 0.7 ± 0.5 kcal mol^–1^, respectively, showing there is little difference in accuracy between the two methods. The Spearman correlations (*r*_*s*_) between the rank order of the predicted and experimental RBFEs have also been calculated, which shows that both methods have a good, and comparable, correlation with experiment. RBFE predictions calculated using MSλD have an *r*_*s*_ of 0.80, while relative FEP predictions have an *r*_*s*_ of 0.70. With this small data set, these differences in *r*_*s*_ are not statistically significant. These results show that MSλD and relative FEP (using the λ window parameters selected from benchmarking) are accurate methods for the prediction of RBFEs and ranking highly active compounds out of a set of congeneric compounds. While the comparison to experiment is similar to the ESMACS (*r*_*s*_ 0.78) and TIES (*r*_*s*_ of 0.92) methods presented by Wan et al.,^21^ MSλD predicts ΔΔ*G* values for the combinatorial set of substituents at each site, and so a larger space of 28 compounds is explored using the four MSλD simulations presented in this work. This is discussed in more detail in the computational expense section.

**Table 2:**
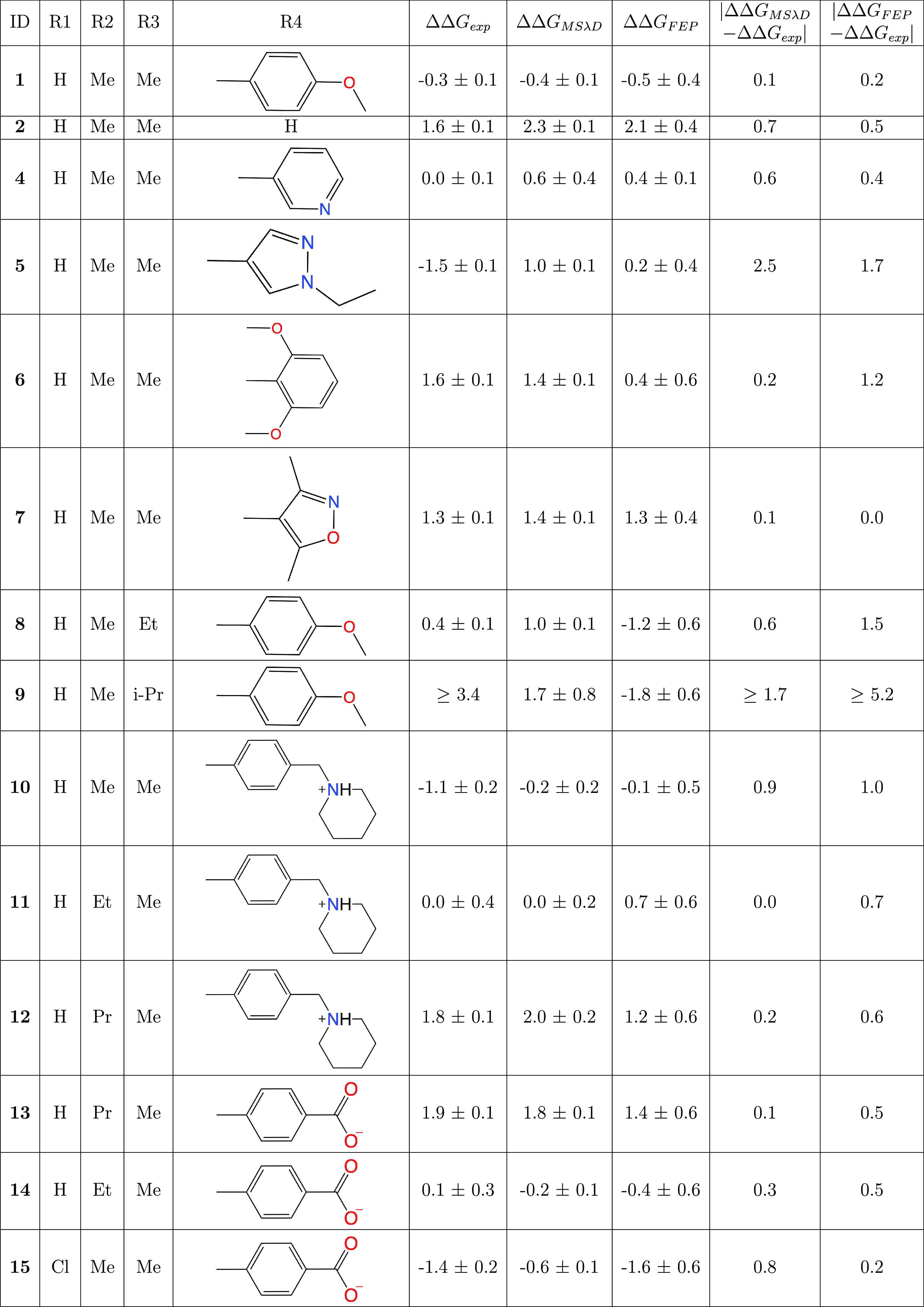
Predictions of Binding Affinity for a Series of BRD4-BD1 Inhibitors Based on a THQ Scaffold (Figure 1)^a^

a Predictions calculated using MSλD and relative FEP are compared to experiment. All free energy differences are shown in kcal mol^–1^.

As discussed previously, all neutral substituents on site 4 were initially included as part of one MSλD calculation. For comparison, the substituents were also split into two calculations. The average RBFE compared to experiment was 1.4 ± 1.4 kcal mol^–1^ when all of the substituents were included in one simulation, while the difference was 0.8 ± 0.8 kcal mol^–1^ when splitting them into two sets of calculations. Furthermore, RBFE predictions obtained from one calculation have an *r*_*s*_ of 0.30, compared to an *r*_*s*_ of 0.84 for the two sets. The increased accuracy when splitting the substituents into two calculations is not surprising. When including all site 4 substituents with a net neutral charge, there are seven possible substituents, which means that all combinations of physically meaningful end points are sampled less during the simulation and less likely to achieve converged results. This is also reflected by the larger uncertainties of the single MSλD simulation, which have an average of 0.4 ± 0.2 kcal mol^–1^ compared to 0.2 ± 0.2 kcal mol^–1^ for the two calculations. Solutions for more accurate predictions in a single simulation could be to use longer simulation times or enhanced sampling methods.^44^ A study by Vilseck et al.^55^ demonstrated that accuracy within 0.8 kcal mol^–1^ can be achieved for perturbation sites with seven substituents when using MSλD with biasing potential replica exchange,^56^ to enhance end-state sampling.

A common limitation to RBFE methods is a lack of reproducibility.^57^ Like all MD-based methods, this arises from the ensemble averaging of macroscopic properties over microscopic states. Therefore, the quality of the predictions relies on how well the microscopic states have been sampled. To address this issue, it is common practice to run multiple independent calculations with different initial velocities and take an average of the free energy changes across the replicas. Uncertainties can be estimated by calculating the standard deviation around the averaged free energies. In our calculations, five replicas were performed for the data collection stages of the MSλD calculations, and three replicas were performed for the relative FEP calculations. Three replicas were chosen for relative FEP due to the significantly higher computational cost associated with this method (discussed in the next section). The uncertainties associated with the predictions were lower for the MSλD calculations, with an average of 0.2 ± 0.2 kcal mol^–1^, compared to an average of 0.5 ± 0.1 kcal mol^–1^ for the relative FEP calculations. Therefore, more reliable estimations of binding affinity are achieved using MSλD, especially when there are more than two sites of perturbation. In these cases, to obtain RBFE values using relative FEP, intermediate transformations are necessary, and the uncertainty accumulates over the two simulations (Free energies and their associated uncertainties for the intermediate calculations can be found in the Supporting Information.). Using MSλD, only one calculation is required, with an uncertainty that is comparable to when there is only one site of perturbation.

#### Outliers

**Figure 8:**
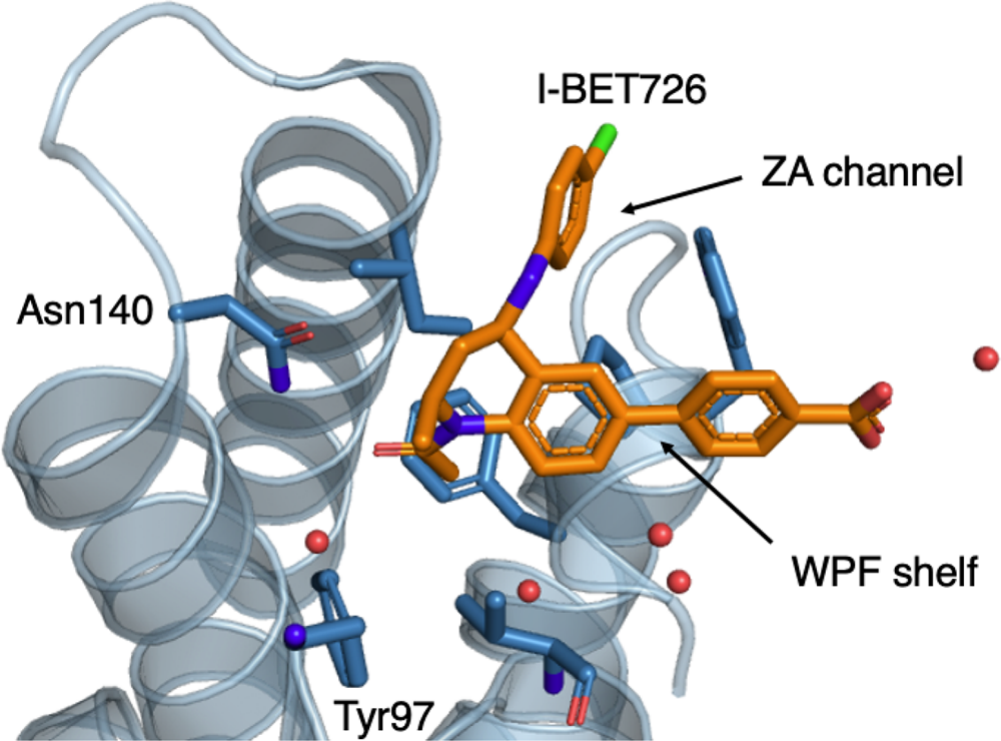
Binding site of BRD4-BD1 with inhibitor I-BET726 bound (PDB 4BJX(58)). I-BET726 (compound **15**) is represented as the stick in orange, the protein is shown as the blue cartoon, and sticks and water molecules are shown as red spheres.

Compound **9** has a pIC_50_ of ≤4.3 and an experimental RBFE of ≥3.4 kcal mol^–1^ with respect to compound **3**, indicating that it has no activity toward BRD4-BD1. The difference in substituents between compound **9** and compound **1**, which has a pIC_50_ of 7.0 ± 0.1, is an isopropyl group at site 2, compared to a methyl group. As noted by Wan et al.,^21^ the position of site 2 occupies a small lipophilic site in the BRD4-BD1 binding pocket, which offers little room for large substituents without structural reorganization. Therefore, we infer that the isopropyl group is too large for this part of the binding pocket. A representative compound in the binding site of BRD4-BD1 is shown in Figure 8. The large discrepancy between the experimental and predicted RBFEs for compound **9** suggests that MSλD and relative FEP methods are less accurate when predicting nonbinders. Additionally, isopropyl is not well represented in the CGenFF force field,^35^ particularly the dihedral angle parameters when attached to an amide, which may also contribute to the deviation from experiment.

A difference larger than 1.5 kcal mol^–1^ from experiment was also found for compound **5** for both RBFE methods. Investigation into the force field parameters and interactions made by the pyrazole derivative at site 4 of compound **5** is ongoing to try and identify a reason for this difference.

### Charge Perturbations

Perturbations that involve a change in net charge of the ligand are difficult to predict with current methods, and the general advice is to avoid them. Cournia et al.^6^ explain that this is due to the PME treatment of long-range interactions, which is likely to introduce an error when changing the net charge of the system. Additionally, care must be taken to ensure that enough time is allowed for the rearrangement of solvent molecules around the ligand when there is a change in charge. Cournia et al. advise that changes in charge should be made to the ligand experimentally, with the results forming the basis for a new series of compounds, with a consistent net charge. Despite this, we believe there was value in investigating how MSλD handles changes in the charge of a ligand, with relative FEP as a comparison, especially as there are few examples in the literature.

**Figure 9:**
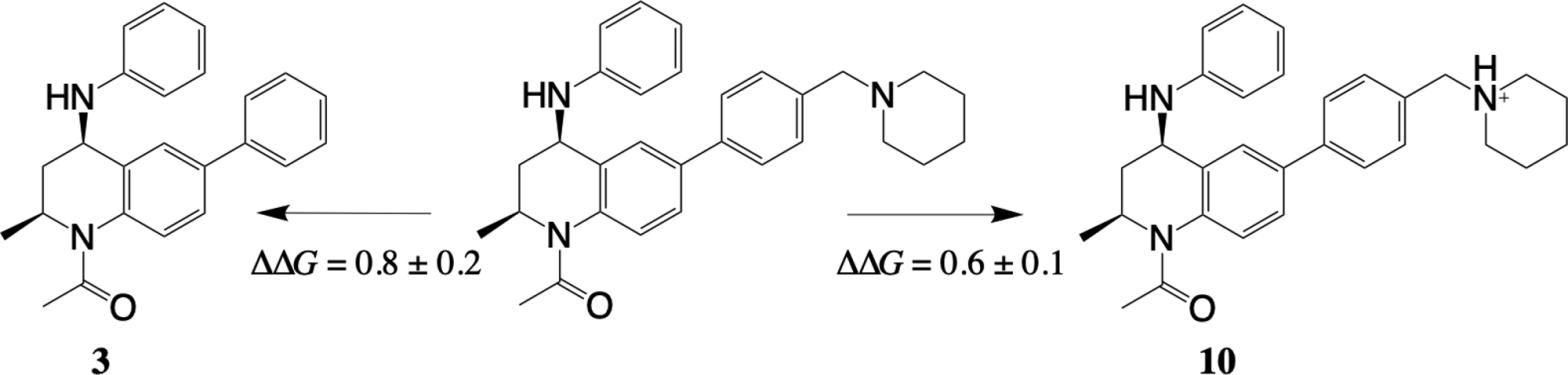
Setup for charge perturbations using MSλD. In this example, the neutral form of compound **10** is used as the reference to calculate the RBFE compared to compound **3** and the protonated form of compound **10**.

As described in the Materials and Methods section, the setup of MSλD calculations was slightly modified for the positively charged piperidine and the negatively charged benzoic acid substituents. A separate MSλD calculation was performed for the positively and negatively charged set of compounds, where the neutral form of the substituent at site 4 was used for each. A phenyl group at site 4 was included in the multiple topology setup so that the RBFE with respect to compound **3** could still be calculated. Figure 9 illustrates the changes in binding free energy calculated for the MSλD perturbation of compound **3** to compound **10**. It should be noted that ethyl and propyl substituents at site 2 were also included so that values of RBFE were obtained for compounds **11** and **12** in the same simulation. Using this approach, the average difference from experiment for the charged compounds was 0.4 ± 0.4 kcal mol^–1^. In comparison, MSλD calculations for the charged substituents without using the neutral reference compound showed an average difference of 0.9 ± 0.3 kcal mol^–1^ from experiment. Therefore, the impressive agreement with experiment shown by our protocol demonstrates that there is a benefit to using a neutral intermediate compound.

Relative FEP predictions for charge perturbations at site 4 also show good agreement with experiment, with an average difference of 0.6 ± 0.3 kcal mol^–1^. The position of site 4 on the THQ scaffold fills the narrow ZA channel in the binding site of BRD4-BD1 and points toward the solvent exposed region (Figure 8). It appears that both MSλD and relative FEP methods accurately predict RBFEs that involve a charge perturbation at this region of the binding pocket.

### Computational Expense

To estimate the computational expense of relative FEP and MSλD calculations applied to this compound series, the simulation time required for each method is calculated. Over four MSλD calculations, 119 ns of ALF and 210 ns of data collection are required. This means the full set of RBFE predictions using MSλD can be calculated with 329 ns of simulation time. This is for predictions where the neutral substituents at site 4 have been split into two calculations, with five replicas performed for each. In contrast, 240 ns of simulation time is required for the RBFE prediction of one pairwise set of compounds using relative FEP, totalling 3360 ns of simulation time for the full set of 14 predictions. Therefore, the MSλD calculations require less simulation time by a factor of ∼10, compared to relative FEP, when considering these 14 compounds. However, as MSλD calculates the RBFE for all combinations of substituents at each site, there is a simulation time saving of a factor of 18, when considering the total molecule space explored. Taking this into account, MSλD provided values for an additional 14 compounds, beyond the 14 presented in Table 2. The compound predicted as having the best binding affinity, relative to compound **3** out of the compounds with experimental data, was compound **15**. This matches experiment, with it having the highest pIC_50_.^21^ From the additional perturbations that MSλD provided, we found that a methyl to ethyl perturbation on site 2 of compound **15** results in an equivalent binding affinity (compound **25** in the Supporting Information). RBFEs for all additional 14 compounds can be found in the Supporting Information. It is also possible for further substituents to be considered at each site with limited additional cost, which would substantially extend the number of compounds evaluated overall.

A nontrivial aspect of relative free energy calculations is the manual time it takes to set up a simulation. These setups are often complicated and prone to human error, and although tools for their automation are being developed, most are in their early stages or are limited to specific simulation programs.^59−61^ Therefore, even with advancements in computational resources and GPU acceleration,^62^ the “human time” required for these calculations often becomes a limitation for the rapid estimation of RBFE for large compound data sets, especially in an academic setting. We have found that for an experienced user and once the initial input scripts have been written, the setup of one MSλD calculation is comparable to the setup of one relative FEP calculation. The difference occurs when considering that one MSλD calculation can provide a large number of binding affinity predictions, whereas a separate simulation is required for every pairwise set of compounds when using relative FEP. Furthermore, the automated MSλD workflow^44^ recently implemented in BIOVIA Discovery Studio and Pipeline Pilot packages^63^ facilitates the needs of MSλD such as setting up multitopologies, which will further accelerate the MSλD method and make it an even more promising alternative to relative FEP for the accurate prediction of ligand binding affinity.

## Conclusions

An investigation into the applicability of MSλD and relative FEP calculations to a series of inhibitors of BRD4-BD1, a prominent therapeutic target, has been carried out. First, benchmarking of relative FEP protocols was performed. Varying numbers of λ windows, equilibration, and data collection periods were used, with the accuracy, uncertainty, and convergence tested for each combination. We found that using 20 λ windows with 0.5 ns of equilibration and 1 ns of data collection was optimal and presented a good compromise between accuracy and efficiency. When applied to the full set of 14 compounds, relative FEP resulted in RBFE predictions with an average accuracy of 0.6 ± 0.6 kcal mol^–1^, when discounting one outlier.

The THQ scaffold has four sites of perturbation, with two substituents at site 1, three at site 2, three at site 3, and nine at site 4. Two of the substituents at site 4 have a charge under physiological conditions and were investigated using separate simulations. To test how well MSλD handles the remaining combinations, all 2 × 3 × 3 × 7 perturbations were considered simultaneously within a single calculation. This resulted in an average accuracy of 1.4 ± 1.4 kcal mol^–1^ and limited correlation between the computed and experimental rank order (*r*_*s*_ = 0.30). MSλD achieved more accurate results when splitting the neutral set of substituents into two independent simulations, with an average accuracy of 0.6 ± 0.7 kcal mol^–1^ for the 14 compounds with experimental values available. A number of perturbations at site 4 involved a change in net charge. Through performing perturbations from the neutral to the charged states of each of these substituents, high accuracy was obtained for these charge changes, with an 0.4 ± 0.4 kcal mol^–1^ average difference from experiment.

MSλD and relative FEP simulations achieved comparable levels of accuracy for this data set. However, the difference lies in the computational cost of the methods. Comparing the amount of simulation time required for each, MSλD required a factor of ∼10 less than relative FEP simulations when considering only those compounds with known free energies but is a factor of ∼18 quicker when the entire molecule space is considered. Furthermore, as a much larger number of compounds can be evaluated using a single MSλD calculation, there is also a saving on manual setup time. Overall, it is clear that MSλD has great potential for the high-throughput prediction of accurate binding affinities in future lead optimization projects.

## Data and Software Availability

Relative FEP calculations were performed using the NAMD 3.0 Alpha simulation software (http://www.ks.uiuc.edu/Research/namd/alpha/3.0alpha). MSLD calculations were performed with the CHARMM molecular simulation package (https://www.charmm.org). All simulation parameters were comprehensively described in the Materials and Methods section, and all relevant molecular structures are available in the Supporting Information.

## Supporting Information Available

Convergence plots for benchmarking of relative FEP protocols, relative binding free energies calculated when including all neutral substituents in single MSλD calculation and relative binding free energies calculated for intermediate relative FEP calculations, and binding affinities calculated using MSλD for all combinations of substituents on scaffold (PDF)
Complex starting structures (ZIP)
SMILES structures of compounds (PDF)

## References

[ref1] Song L. F.; Merz K. M. Evolution of Alchemical Free Energy Methods in Drug Discovery. J. Chem. Inf. Model. 2020, 60, 5308–5318. 10.1021/acs.jcim.0c00547. 32818371

[ref2] Zwanzig R. W. High-Temperature Equation of State by a Perturbation Method. I. Nonpolar Gases. J. Chem. Phys. 1954, 22, 1420–1426. 10.1063/1.1740409.

[ref3] Bash P. A.; Singh U. C.; Brown F. K.; Langridge R.; Kollman P. A. Calculation of the Relative Change in Binding Free Energy of a Protein-Inhibitor Complex. Science 1987, 235, 574–576. 10.1126/science.3810157. 3810157

[ref4] Straatsma T. P.; Berendsen H. J. Free Energy of Ionic Hydration: Analysis of a Thermodynamic Integration Technique to Evaluate Free Energy Differences by Molecular Dynamics Simulations. J. Chem. Phys. 1988, 89, 5876–5886. 10.1063/1.455539.

[ref5] Straatsma T. P.; McCammon J. A. Multiconfiguration Thermodynamic Integration. J. Chem. Phys. 1991, 95, 1175–1188. 10.1063/1.461148.

[ref6] Cournia Z.; Allen B. K.; Beuming T.; Pearlman D. A.; Radak B. K.; Sherman W. Rigorous Free Energy Simulations in Virtual Screening. J. Chem. Inf. Model. 2020, 60, 4153–4169. 10.1021/acs.jcim.0c00116. 32539386

[ref7] Kuhn M.; Firth-Clark S.; Tosco P.; Mey A. S.; MacKey M.; Michel J. Assessment of Binding Affinity via Alchemical Free-Energy Calculations. J. Chem. Inf. Model. 2020, 60, 3120–3130. 10.1021/acs.jcim.0c00165. 32437145

[ref8] Knight J. L.; Brooks C. L. III λ-Dynamics Free Energy Simulation Methods. J. Comput. Chem. 2009, 30, 1692–1700. 10.1002/jcc.21295. 19421993 PMC2730455

[ref9] Kong X.; Brooks C. L. III λ-Dynamics: A New Approach to Free Energy Calculations. J. Chem. Phys. 1996, 105, 2414–2423. 10.1063/1.472109.

[ref10] Hayes R. L.; Armacost K. A.; Vilseck J. Z.; Brooks C. L. III Adaptive Landscape Flattening Accelerates Sampling of Alchemical Space in Multisite λ Dynamics. J. Phys. Chem. B 2017, 121, 3626–3635. 10.1021/acs.jpcb.6b09656. 28112940 PMC5824625

[ref11] Cheng Y.; He C.; Wang M.; Ma X.; Mo F.; Yang S.; Han J.; Wei X. Targeting Epigenetic Regulators for Cancer Therapy: Mechanisms and Advances in Clinical Trials. Signal Transduct Target Ther 2019, 4, 62 10.1038/s41392-019-0095-0. 31871779 PMC6915746

[ref12] Padmanabhan B.; Mathur S.; Manjula R.; Tripathi S. Bromodomain and Extra-Terminal (BET) Family Proteins: New Therapeutic Targets in Major Diseases. J. Biosci 2016, 41, 295–311. 10.1007/s12038-016-9600-6. 27240990

[ref13] Korb E.; Herre M.; Zucker-Scharff I.; Darnell R. B.; Allis C. D. BET Protein BRD4 Activates Transcription in Neurons and BET Inhibitor JQ1 Blocks Memory in Mice. Nat. Neurosci. 2015, 18, 1464–1473. 10.1038/nn.4095. 26301327 PMC4752120

[ref14] Belkina A. C.; Denis G. V. BET Domain Co-Regulators in Obesity, Inflammation and Cancer. Nat. Rev. Cancer 2012, 12, 465–477. 10.1038/nrc3256. 22722403 PMC3934568

[ref15] Filippakopoulos P.; Qi J.; Picaud S.; Shen Y.; Smith W. B.; Fedorov O.; Morse E. M.; Keates T.; Hickman T. T.; Felletar I.; Philpott M.; Munro S.; McKeown M. R.; Wang Y.; Christie A. L.; West N.; Cameron M. J.; Schwartz B.; Heightman T. D.; Thangue N. L.; French C. A.; Wiest O.; Kung A. L.; Knapp S.; Bradner J. E. Selective Inhibition of BET Bromodomains. Nature 2010, 468, 1067–1073. 10.1038/nature09504. 20871596 PMC3010259

[ref16] Chen L. F.; Mu Y.; Greene W. C. Acetylation of RelA at Discrete Sites Regulates Distinct Nuclear Functions of NF-κB. EMBO J. 2002, 21, 6539–6548. 10.1093/emboj/cdf660. 12456660 PMC136963

[ref17] Gilan O.; Rioja I.; Knezevic K.; Bell M. J.; Yeung M. M.; Harker N. R.; Lam E. Y.; Chung C.; Bamborough P.; Petretich M.; Urh M.; Atkinson S. J.; Bassil A. K.; Roberts E. J.; Vassiliadis D.; Burr M. L.; Preston A. G.; Wellaway C.; Werner T.; Gray J. R.; Michon A. M.; Gobbetti T.; Kumar V.; Soden P. E.; Haynes A.; Vappiani J.; Tough D. F.; Taylor S.; Dawson S. J.; Bantscheff M.; Lindon M.; Drewes G.; Demont E. H.; Daniels D. L.; Grandi P.; Prinjha R. K.; Dawson M. A. Selective Targeting of BD1 and BD2 of the BET Proteins in Cancer and Immunoinflammation. Science 2020, 368, 387–394. 10.1126/science.aaz8455. 32193360 PMC7610820

[ref18] Jones K. L.; Beaumont D. M.; Bernard S. G.; Bit R. A.; Campbell S. P.; wa Chung C.; Cutler L.; Demont E. H.; Dennis K.; Gordon L.; Gray J. R.; Haase M. V.; Lewis A. J.; McCleary S.; Mitchell D. J.; Moore S. M.; Parr N.; Robb O. J.; Smithers N.; Soden P. E.; Suckling C. J.; Taylor S.; Walker A. L.; Watson R. J.; Prinjha R. K. Discovery of a Novel Bromodomain and Extra Terminal Domain (BET) Protein Inhibitor, I-BET282E, Suitable for Clinical Progression. J. Med. Chem. 2021, 64, 12200–12227. 10.1021/acs.jmedchem.1c00855. 34387088

[ref19] Liu Z.; Wang P.; Chen H.; Wold E. A.; Tian B.; Brasier A. R.; Zhou J. Drug Discovery Targeting Bromodomain-Containing Protein 4. J. Med. Chem. 2017, 60, 4533–4558. 10.1021/acs.jmedchem.6b01761. 28195723 PMC5464988

[ref20] Aldeghi M.; Heifetz A.; Bodkin M. J.; Knapp S.; Biggin P. C. Accurate Calculation of the Absolute Free Energy of Binding for Drug Molecules. Chem. Sci. 2016, 7, 207–218. 10.1039/C5SC02678D. 26798447 PMC4700411

[ref21] Wan S.; Bhati A. P.; Zasada S. J.; Wall I.; Green D.; Bamborough P.; Coveney P. V. Rapid and Reliable Binding Affinity Prediction of Bromodomain Inhibitors: A Computational Study. J. Chem. Theory Comput. 2017, 13, 784–795. 10.1021/acs.jctc.6b00794. 28005370 PMC5312866

[ref22] Wan S.; Knapp B.; Wright D. W.; Deane C. M.; Coveney P. V. Rapid, Precise, and Reproducible Prediction of Peptide-MHC Binding Affinities from Molecular Dynamics That Correlate Well with Experiment. J. Chem. Theory Comput. 2015, 11, 3346–3356. 10.1021/acs.jctc.5b00179. 26575768

[ref23] Bhati A. P.; Wan S.; Wright D. W.; Coveney P. V. Rapid, Accurate, Precise, and Reliable Relative Free Energy Prediction using Ensemble Based Thermodynamic Integration. J. Chem. Theory Comput. 2017, 13, 210–222. 10.1021/acs.jctc.6b00979. 27997169

[ref24] Gapsys V.; Yildirim A.; Aldeghi M.; Khalak Y.; van der Spoel D.; de Groot B. L. Accurate Absolute Free Energies for Ligand–Protein Binding Based on Non-Equilibrium Approaches. Commun. Chem. 2021, 4, 61 10.1038/s42004-021-00498-y. PMC9814727 36697634

[ref25] Knight J. L.; Brooks C. L. III Applying Efficient Implicit Nongeometric Constraints in Alchemical Free Energy Simulations. J. Comput. Chem. 2011, 32, 3423–3432. 10.1002/jcc.21921. 21919014 PMC3196384

[ref26] Hayes R. L.; Armacost K. A.; Vilseck J. Z.; Brooks C. L. III Adaptive Landscape Flattening Accelerates Sampling of Alchemical Space in Multisite λ Dynamics. J. Chem. Phys. B 2017, 121, 3626–3635. 10.1021/acs.jpcb.6b09656. PMC5824625 28112940

[ref27] Jung M.; Philpott M.; Müller S.; Schulze J.; Badock V.; Eberspächer U.; Moosmayer D.; Bader B.; Schmees N.; Fernández-Montalván A.; Haendler B. Affinity Map of Bromodomain Protein 4 (BRD4) Interactions with the Histone H4 Tail and the Small Molecule Inhibitor JQ1. J. Biol. Chem. 2014, 289, 9304–9319. 10.1074/jbc.M113.523019. 24497639 PMC3979416

[ref28] Crawford T. D.; Tsui V.; Flynn E. M.; Wang S.; Taylor A. M.; Côté A.; Audia J. E.; Beresini M. H.; Burdick D. J.; Cummings R.; Dakin L. A.; Duplessis M.; Good A. C.; Hewitt M. C.; Huang H. R.; Jayaram H.; Kiefer J. R.; Jiang Y.; Murray J.; Nasveschuk C. G.; Pardo E.; Poy F.; Romero F. A.; Tang Y.; Wang J.; Xu Z.; Zawadzke L. E.; Zhu X.; Albrecht B. K.; Magnuson S. R.; Bellon S.; Cochran A. G. Diving into the Water: Inducible Binding Conformations for BRD4, TAF1(2), BRD9, and CECR2 Bromodomains. J. Med. Chem. 2016, 59, 5391–5402. 10.1021/acs.jmedchem.6b00264. 27219867

[ref29] Filippakopoulos P.; Knapp S. Targeting Bromodomains: Epigenetic Readers of Lysine Acetylation. Nat. Rev. Drug Discovery 2014, 13, 337–356. 10.1038/nrd4286. 24751816

[ref30] Zhong H.; Wang Z.; Wang X.; Liu H.; Li D.; Liu H.; Yao X.; Hou T. Importance of a Crystalline Water Network in Docking-Based Virtual Screening: a Case Study of BRD4. Phys. Chem. Chem. Phys. 2019, 21, 25276–25289. 10.1039/C9CP04290C. 31701109

[ref31] Guest E. E.; Pickett S. D.; Hirst J. D. Structural Variation of Protein–Ligand Complexes of the First Bromodomain of BRD4. Org. Biomol. Chem. 2021, 19, 5632–5641. 10.1039/D1OB00658D. 34105560

[ref32] McGann M. FRED Pose Prediction and Virtual Screening Accuracy. J. Chem. Inf. Model. 2011, 51, 578–596. 10.1021/ci100436p. 21323318

[ref33] Hawkins P. C.; Skillman A. G.; Warren G. L.; Ellingson B. A.; Stahl M. T. Conformer Generation with OMEGA: Algorithm and Validation using High Quality Structures from the Protein Databank and Cambridge Structural Database. J. Chem. Inf. Model. 2010, 50, 572–584. 10.1021/ci100031x. 20235588 PMC2859685

[ref34] Halgren T. A. MMFF VI. MMFF94s Option for Energy Minimization Studies. J. Comput. Chem. 1999, 20, 720–729. 10.1002/(SICI)1096-987X(199905)20:7<720::AID-JCC7>3.0.CO;2-X. 34376030

[ref35] Vanommeslaeghe K.; Hatcher E.; Acharya C.; Kundu S.; Zhong S.; Shim J.; Darian E.; Guvench O.; Lopes P.; Vorobyov I.; MacKerell A. D. CHARMM General Force Field: A Force Field for Drug-Like Molecules Compatible with the CHARMM All-Atom Additive Biological Force Fields. J. Comput. Chem. 2009, 31, 671–690. 10.1002/jcc.21367. PMC2888302 19575467

[ref36] Knight J. L.; Brooks C. L. III Multisite λ-Dynamics for Simulated Structure-Activity Relationship Studies. J. Chem. Theory Comput. 2011, 7, 2728–2739. 10.1021/ct200444f. 22125476 PMC3223982

[ref37] Brooks B. R.; Brooks C. L. III; A D. M. Jr.; Nilsson L.; Petrella R. J.; Roux B.; Won Y.; Archontis G.; Bartels C.; Boresch S.; Caflisch A.; Caves L.; Cui Q.; Dinner A. R.; Feig M.; Fischer S.; Gao J.; Hodoscek M.; Im W.; Kuczera K.; Lazaridis T.; Ma J.; Ovchinnikov V.; Paci E.; Pastor R. W.; Post C. B.; Pu J. Z.; Schaefer M.; Tidor B.; Venable R. M.; Woodcock H. L.; Wu X.; Yang W.; York D. M.; Karplus M. CHARMM: The Biomolecular Simulation Program. J. Comput. Chem. 2009, 30, 1545–1614. 10.1002/jcc.21287. 19444816 PMC2810661

[ref38] Jorgensen W. L.; Chandrasekhar J.; Madura J. D.; Impey R. W.; Klein M. L. Comparison of Simple Potential Functions for Simulating Liquid Water. J. Chem. Phys. 1983, 79, 926–935. 10.1063/1.445869.

[ref39] Brooks B.; Bruccoleri R.; Olafson B.; States D.; Swaminathan S.; Karplus M. CHARMM: A Program for Macromolecular Energy, Minimization, and Dynamics Calculations. J. Comput. Chem. 1983, 4, 187–217. 10.1002/jcc.540040211.

[ref40] Hynninen A. P.; Crowley M. F. New Faster CHARMM Molecular Dynamics Engine. J. Comput. Chem. 2014, 35, 406–413. 10.1002/jcc.23501. 24302199 PMC3966901

[ref41] Hoover W. G. Canonical Dynamics: Equilibrium Phase-Space Distributions. Phys. Rev. A 1985, 31, 1695–1697. 10.1103/PhysRevA.31.1695. 9895674

[ref42] Feller S. E.; Zhang Y.; Pastor R. W.; Brooks B. R. Constant Pressure Molecular Dynamics Simulation: The Langevin Piston Method. J. Comput. Phys. 1995, 103, 4613–4621. 10.1063/1.470648.

[ref43] Ryckaert J.-P.; Ciccotti G.; Berendsen H. J. C. Numerical Integration of the Cartesian Equations of Motion of a System with Constraints: Molecular Dynamics of n-Alkanes. J. Comput. Phys. 1977, 23, 327–341. 10.1016/0021-9991(77)90098-5.

[ref44] Raman E. P.; Paul T. J.; Hayes R. L.; Brooks C. L. III Automated, Accurate, and Scalable Relative Protein-Ligand Binding Free-Energy Calculations Using Lambda Dynamics. J. Chem. Theory Comput. 2020, 16, 7895–7914. 10.1021/acs.jctc.0c00830. 33201701 PMC7814773

[ref45] Zacharias M.; Straatsma T. P.; McCammon J. A. Separation-Shifted Scaling, a New Scaling Method for Lennard-Jones Interactions in Thermodynamic Integration. J. Chem. Phys. 1994, 100, 9025–9031. 10.1063/1.466707.

[ref46] Beutler T. C.; Mark A. E.; van Schaik R. C.; Gerber P. R.; van Gunsteren W. F. Avoiding Singularities and Numerical Instabilities in Free Energy Calculations Based on Molecular Simulations. Chem. Phys. Lett. 1994, 222, 529–539. 10.1016/0009-2614(94)00397-1.

[ref47] Jo S.; Kim T.; Iyer V. G.; Im W. CHARMM-GUI: A Web-Based Graphical User Interface for CHARMM. J. Comput. Chem. 2008, 29, 1859–1865. 10.1002/jcc.20945. 18351591

[ref48] Best R. B.; Zhu X.; Shim J.; Lopes P. E. M.; Mittal J.; Feig M.; MacKerell A. D. Optimization of the Additive CHARMM All-Atom Protein Force Field Targeting Improved Sampling of the Backbone *ϕ, ψ* and Side-Chain χ1 and χ2 Dihedral Angles. J. Chem. Theory Comput. 2012, 8, 3257–3273. 10.1021/ct300400x. 23341755 PMC3549273

[ref50] Phillips J. C.; Braun R.; Wang W.; Gumbart J.; Tajkhorshid E.; Villa E.; Chipot C.; Skeel R. D.; Kalé L.; Schulten K. Scalable Molecular Dynamics with NAMD. J. Comput. Chem. 2005, 26, 1781–802. 10.1002/jcc.20289. 16222654 PMC2486339

[ref51] Feller S. E.; Pastor R. W.; Rojnuckarin A.; Bogusz S.; Brooks B. R. Effect of Electrostatic Force Truncation on Interfacial and Transport Properties of Water. J. Phys. Chem. 1996, 100, 17011–17020. 10.1021/jp9614658.

[ref52] Bennett C. H. Efficient Estimation of Free Energy Differences from Monte Carlo Data. J. Comput. Phys. 1976, 22, 245–268. 10.1016/0021-9991(76)90078-4.

[ref53] Liu P.; Dehez F.; Cai W.; Chipot C. A Toolkit for the Analysis of Free-Energy Perturbation Calculations. J. Chem. Theory Comput. 2012, 8, 2606–2616. 10.1021/ct300242f. 26592106

[ref54] Klimovich P. V.; Shirts M. R.; Mobley D. L. Guidelines for the Analysis of Free Energy Calculations. J. Comput.-Aided Mol. Des. 2015, 29, 397–411. 10.1007/s10822-015-9840-9. 25808134 PMC4420631

[ref55] Vilseck J. Z.; Sohail N.; Hayes R. L.; Brooks C. L. III Overcoming Challenging Substituent Perturbations with Multisite λ-Dynamics: A Case Study Targeting β-Secretase 1. J. Phys. Chem. Lett. 2019, 10, 4875–4880. 10.1021/acs.jpclett.9b02004. 31386370 PMC7015761

[ref56] Armacost K. A.; Goh G. B.; Brooks C. L. III Biasing Potential Replica Exchange Multisite λ-Dynamics for Efficient Free Energy Calculations. J. Chem. Theory Comput. 2015, 11, 1267–1277. 10.1021/ct500894k. 26579773 PMC4731093

[ref57] Bhati A. P.; Wan S.; Hu Y.; Sherborne B.; Coveney P. V. Uncertainty Quantification in Alchemical Free Energy Methods. J. Chem. Theory Comput. 2018, 14, 2867–2880. 10.1021/acs.jctc.7b01143. 29678106 PMC6095638

[ref58] Wyce A.; Ganji G.; Smitheman K. N.; Chung C.; Korenchuk S.; Bai Y.; Barbash O.; Le B. C.; Craggs P. D.; McCabe M. T.; Kennedy-Wilson K. M.; Sanchez L. V.; Gosmini R. L.; Parr N.; McHugh C. F.; Dhanak D.; Prinjha R. K.; Auger K. R.; Tummino P. J. BET Inhibition Silences Expression of MYCN and BCL2 and Induces Cytotoxicity in Neuroblastoma Tumor Models. PLoS One 2013, 8, e72967 10.1371/journal.pone.0072967. 24009722 PMC3751846

[ref59] Loeffler H. H.; Michel J.; Woods C. FESetup: Automating Setup for Alchemical Free Energy Simulations. J. Chem. Inf. Model. 2015, 55, 2485–2490. 10.1021/acs.jcim.5b00368. 26544598

[ref60] Jespers W.; Esguerra M.; Åqvist J.; Gutiérrez-De-Terán H. Qligfep: An Automated Workflow for Small Molecule Free Energy Calculations in Q. J. Cheminf. 2019, 11, 26 10.1186/s13321-019-0348-5. PMC6444553 30941533

[ref61] Carvalho Martins L.; Cino E. A.; Ferreira R. S. PyAutoFEP: An Automated Free Energy Perturbation Workflow for GROMACS Integrating Enhanced Sampling Methods. J. Chem. Theory Comput. 2021, 17, 4262–4273. 10.1021/acs.jctc.1c00194. 34142828

[ref62] Phillips J. C.; Hardy D. J.; Maia J. D.; Stone J. E.; Ribeiro J. V.; Bernardi R. C.; Buch R.; Fiorin G.; Hénin J.; Jiang W.; McGreevy R.; Melo M. C.; Radak B. K.; Skeel R. D.; Singharoy A.; Wang Y.; Roux B.; Aksimentiev A.; Luthey-Schulten Z.; Kalé L. V.; Schulten K.; Chipot C.; Tajkhorshid E. Scalable Molecular Dynamics on CPU and GPU Architectures with NAMD. J. Chem. Phys. 2020, 153, 044130 10.1063/5.0014475. 32752662 PMC7395834

[ref63] BIOVIA Discovery Studio Modeling Environment. https://www.3ds.com/products-services/biovia/products/molecular-modeling-simulation/biovia-discovery-studio/ (accessed 2022-01-05).

