# Supplementary material for: Alchemical Free Energy Methods Applied to Complexes of the First Bromodomain of BRD4
Source: J Chem Inf Model. 2022 Mar 8;62(6):1458–70. doi: 10.1021/acs.jcim.1c01229 (PMC9098113; doi:10.1021/acs.jcim.1c01229)
Supplement: Supplementary file 1 — ci1c01229_si_001.pdf [file ci1c01229_si_001.pdf]

Supporting Information - Alchemical Free Energy Methods Applied to Complexes of the First Bromodomain of BRD4

Ellen E. Guest,[†] Luis F. Cervantes,[‡] Stephen D. Pickett,[¶] Charles L. Brooks III,[‡] and Jonathan D. Hirst[*,†]

†*School of Chemistry, University of Nottingham, University Park, Nottingham, NG7 2RD, U.K.*

‡*Department of Chemistry, University of Michigan, Ann Arbor, Michigan, 48109, U.S.A.*

¶*GlaxoSmithKline RD Pharmaceuticals, Computational Chemistry, Stevenage, SG1 2NY, U.K.*

E-mail: jonathan.hirst@nottingham.ac.uk

Charge re-normalisation

Charge re-normalisation is a recently developed approach for partitioning partial atomic charges in a manner to facilitate MSλD setup and sampling. Described in detail in a forthcoming publication, this approach produces negligible shifts in the overall charge distribution and thus only introduces negligible errors into the MSλD free energy results, within statistical noise.

Convergence assessment of relative FEP simulations for the calculation of binding free energies for BRD4-ligand complexes

We describe the benchmarking of relative FEP simulations with varying numbers of λ windows, equilibration length and data collection length. The following plots show the convergence of the binding free energy with each combination of parameters tested. The forward (purple lines) and the reverse (green lines) simulation time series are shown and the horizontal shaded bar indicates ± 0.5 kcal mol^{-1} of the final value.

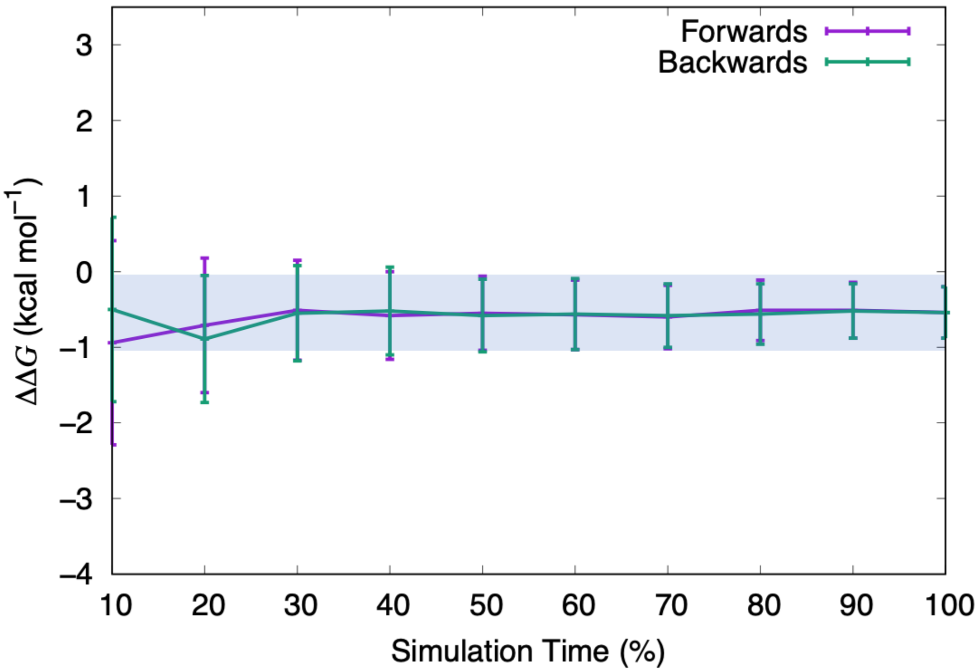

Figure S1: Convergence assessment of a relative FEP simulation using 25 λ windows with 2 ns of equilibration and 1 ns of data collection.

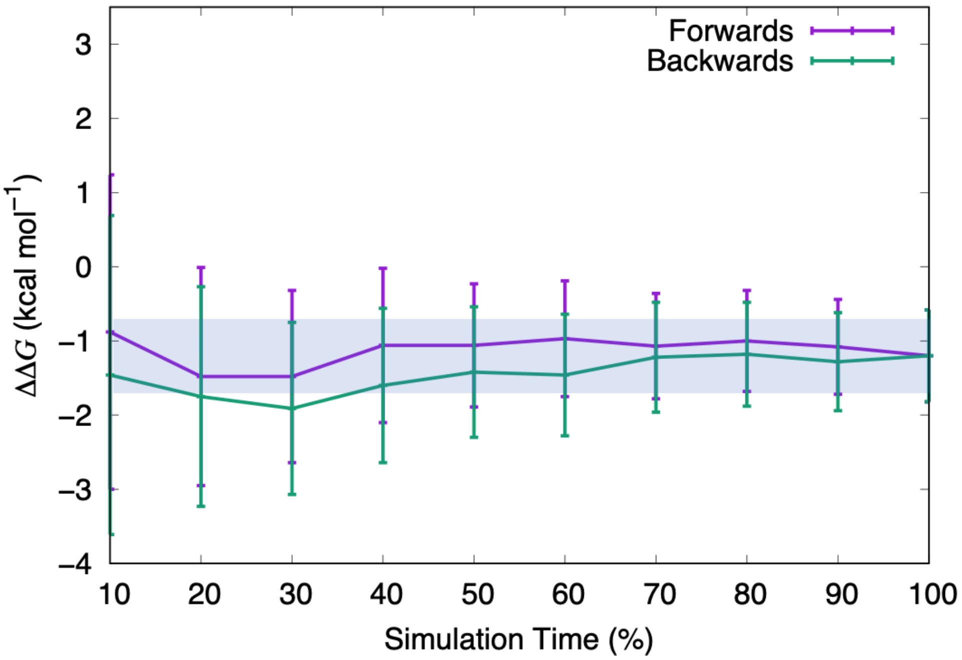

Figure S2: Convergence assessment of a relative FEP simulation using 10 λ windows with 2 ns of equilibration and 1 ns of data collection.

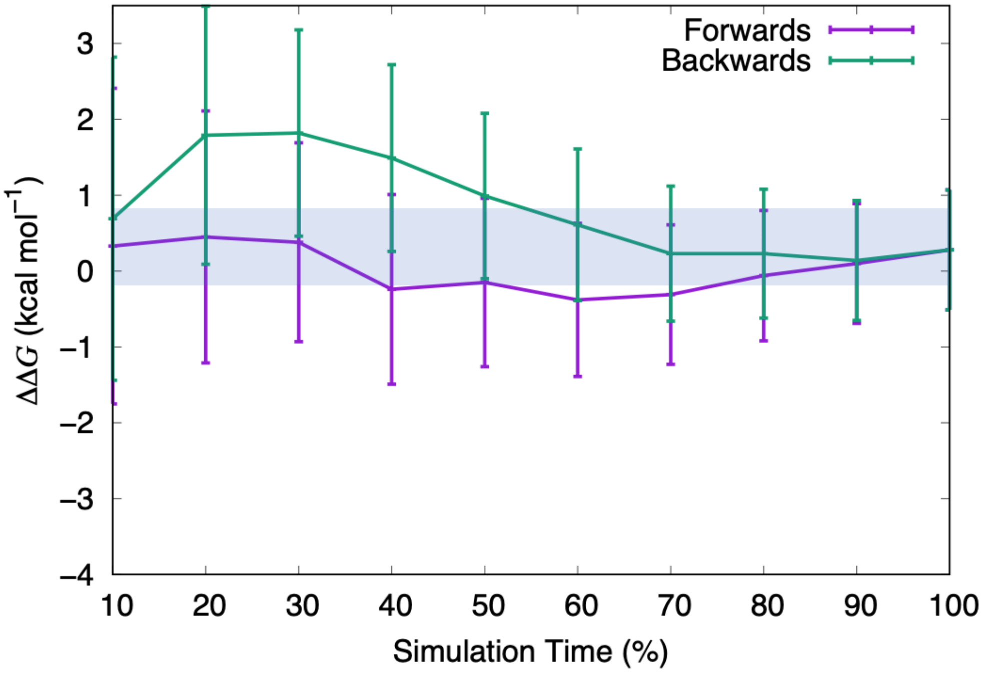

Figure S3: Convergence assessment of a relative FEP simulation using 8 λ windows with 2 ns of equilibration and 1 ns of data collection.

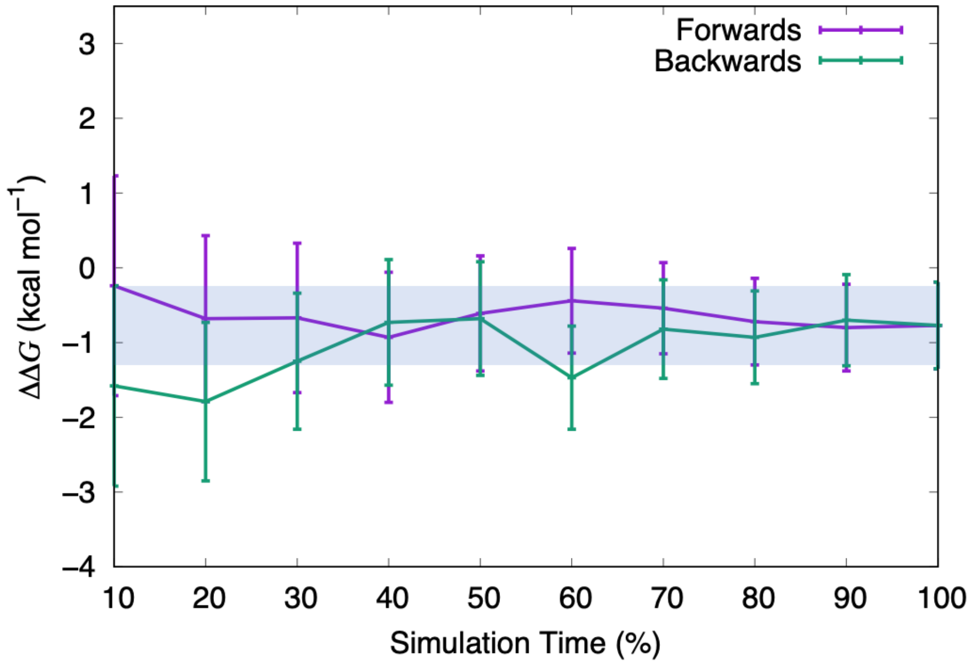

Figure S4: Convergence assessment of a relative FEP simulation using 20 λ windows with 2 ns of equilibration and 0.5 ns of data collection.

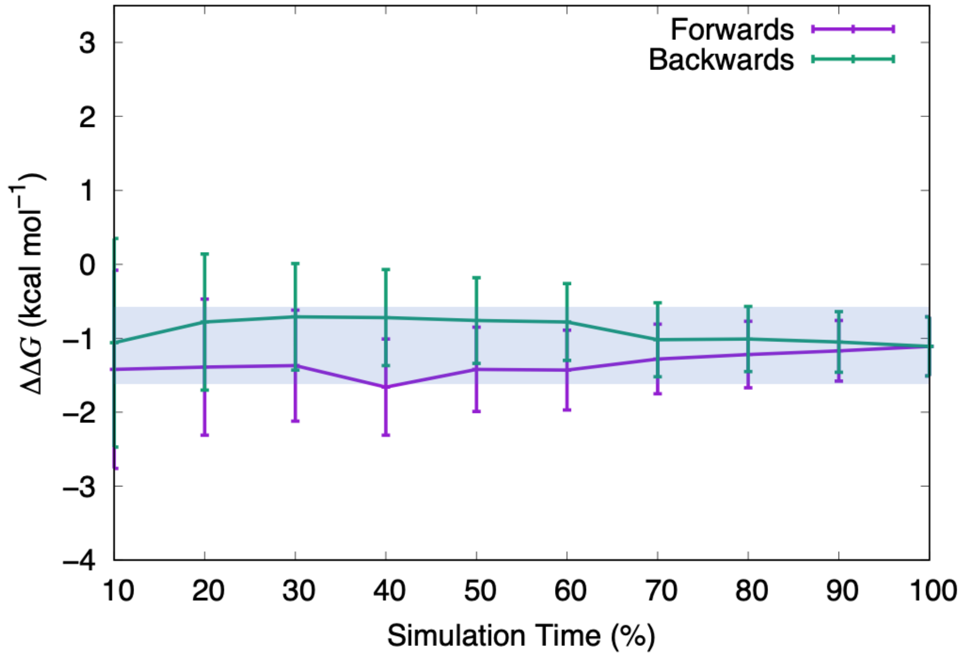

Figure S5: Convergence assessment of a relative FEP simulation using 20 λ windows with 1 ns of equilibration and 1 ns of data collection.

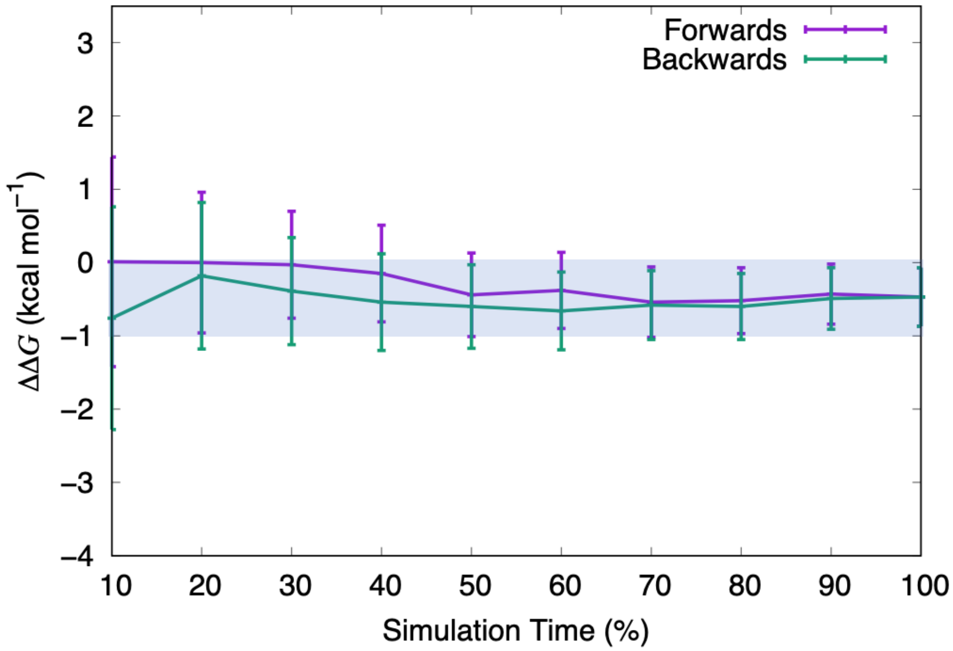

Figure S6: Convergence assessment of a relative FEP simulation using 20 λ windows with 0.5 ns of equilibration and 1 ns of data collection.

RBFE predictions of BRD4-BD1 inhibitors with a net neutral charge calculated using a single MSλD simulation

Predicted relative binding affinities for compounds with a net neutral charge, calculated using a single MSλD simulation are shown below. The reference compound for each perturbation is compound **3**. In the main text, results are presented for compounds when they were split into two MSλD simulations, which achieved more accurate results compared to the ones presented below.

Table S1: RBFE predictions for a series of BRD4-BD1 inhibitors calculated using a single MSλD simulation. Relative free energy changes are shown in kcal mol^{-1}. R positions correspond to Figure 1 in the main text.

ID	R1	R2	R3	R4	$\Delta\Delta G_{exp}$	$\Delta\Delta G_{MS\lambda D}$	$\lvert\Delta\Delta G_{MS\lambda D} - \Delta\Delta G_{exp}\rvert$
**1**	H	Me	Me	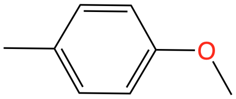	-0.3 ± 0.1	0.3 ± 0.2	0.6
**2**	H	Me	Me	H	1.6 ± 0.1	1.1 ± 0.7	0.5
**4**	H	Me	Me	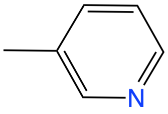	0.0 ± 0.1	0.7 ± 0.4	0.7
**5**	H	Me	Me	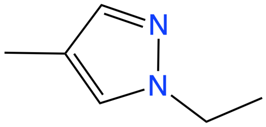	-1.5 ± 0.1	2.1 ± 0.4	3.6
**6**	H	Me	Me	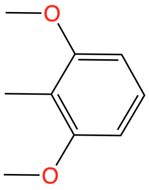	1.6 ± 0.1	0.2 ± 0.4	1.4
**7**	H	Me	Me	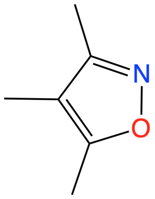	1.3 ± 0.1	1.2 ± 0.2	0.1
**8**	H	Me	Et	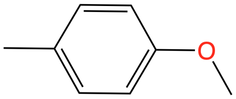	0.4 ± 0.1	-0.2 ± 0.4	0.6
**9**	H	Me	i-Pr	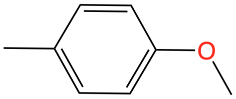	≥ 3.4	-0.1 ± 0.3	≥ 3.5

RBFE predictions for intermediate compounds using relative FEP

Relative FEP simulations are used to calculate relative binding affinities for a series of BRD4-BD1 inhibitors. A few of these perturbations involved changing substituents on more than one attachment point of the scaffold. Therefore, intermediate transformations were required. The results of which are shown below. The structure of compound **15h** is shown in Figure S7.

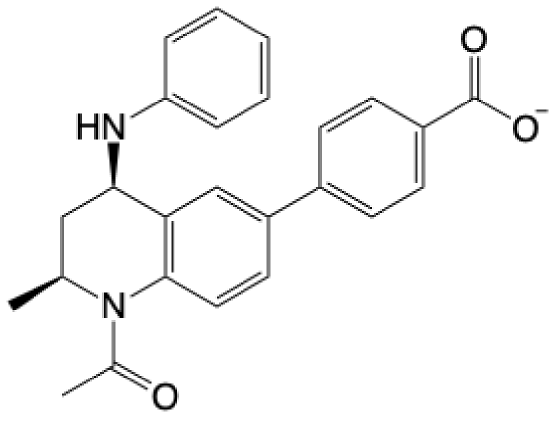

15h

Figure S7: The structure of compound **15h**, which is used as an intermediate FEP compound for the calculation of RBFE for compounds **13**, **14** and **15**, with respect to compound **3**.

Table S2: RBFE predictions for intermediate compounds using relative FEP. Compound numbers correspond to those in Table 2 in the main body of the text.

Transformation	$\Delta\Delta G_{calc}$ (kcal mol^{-1})	Uncertainty (kcal mol^{-1})
**3 → 10**	-0.1	0.5
**10 → 11**	0.7	0.2
**10 → 12**	1.9	0.2
**3 → 15h**	-0.7	0.6
**15h → 13**	2.1	0.3
**15h → 14**	0.3	0.2
**15h → 15**	-0.9	0.2

RBFE predictions of all compounds explored using MSλD

MSλD calculates the RBFE for all combinations of the substituents provided at each site. This means 25 additional compounds, beyond the 14 with known experimental values, were evaluated using MSλD. Table S3 and Figure S8 show the RBFE for all compounds explored using MSλD. Experimental RBFE values are available for compounds **1** to **15**.[1] Binding free energies for compound **3** are not shown as this is the reference compound for the relative RBFE values. Compound **16** from the original dataset is not studied as it is a stereo-isomer of compound **1**.[1] Predicting binding free energies for isomers is beyond the scope of this work. No experimental data is available for compounds **17** to **30**.

Table S3: RBFE predictions for a series of BRD4-BD1 inhibitors calculated using MSλD. Relative free energy changes are shown in kcal mol^{-1}. R positions correspond to Figure 1 in the main text.

ID	R1	R2	R3	R4	$\Delta\Delta G_{MS\lambda D}$
**17**	H	Me	Et	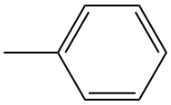	1.0 ± 0.1
**18**	H	Me	i-Pr	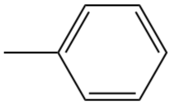	2.0 ± 0.1
**19**	H	Me	Et	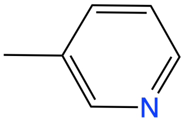	1.7 ± 0.5
**20**	H	Me	i-Pr	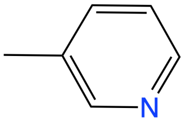	2.5 ± 0.7
**21**	H	Me	Et	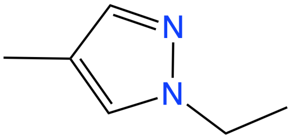	1.7 ± 0.2
**22**	H	Me	i-Pr	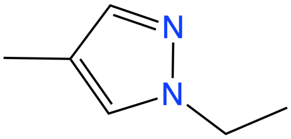	2.3 ± 0.2

ID	R1	R2	R3	R4	$\Delta\Delta G_{MS\lambda D}$
**23**	H	Me	Me	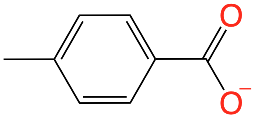	-0.3 ± 0.1
**24**	Cl	Pr	Me	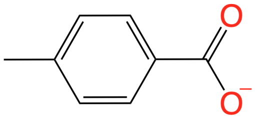	1.7 ± 0.1
**25**	Cl	Et	Me	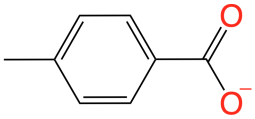	-0.6 ± 0.1
**26**	H	Pr	Me	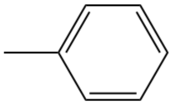	2.1 ± 0.1
**27**	H	Et	Me	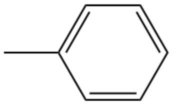	0.1 ± 0.1
**28**	Cl	Me	Me	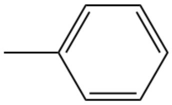	-0.2 ± 0.1
**29**	Cl	Pr	Me	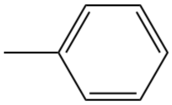	1.9 ± 0.1
**30**	Cl	Et	Me	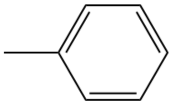	-0.2 ± 0.1

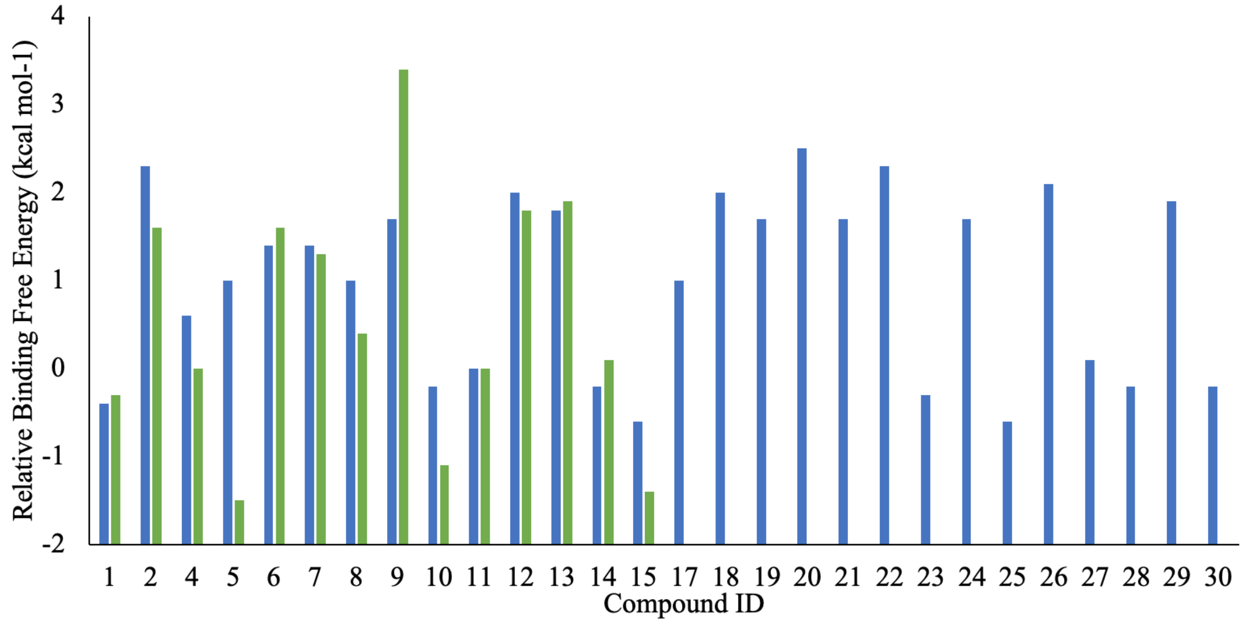

Figure S8: RBFE values for all compounds calculated using MSλD simulations. Predicted values are shown as blue bars. Experimental values, for those compounds with data available, are shown as green bars.

Sampling of site 4-phenyl dihedral angles

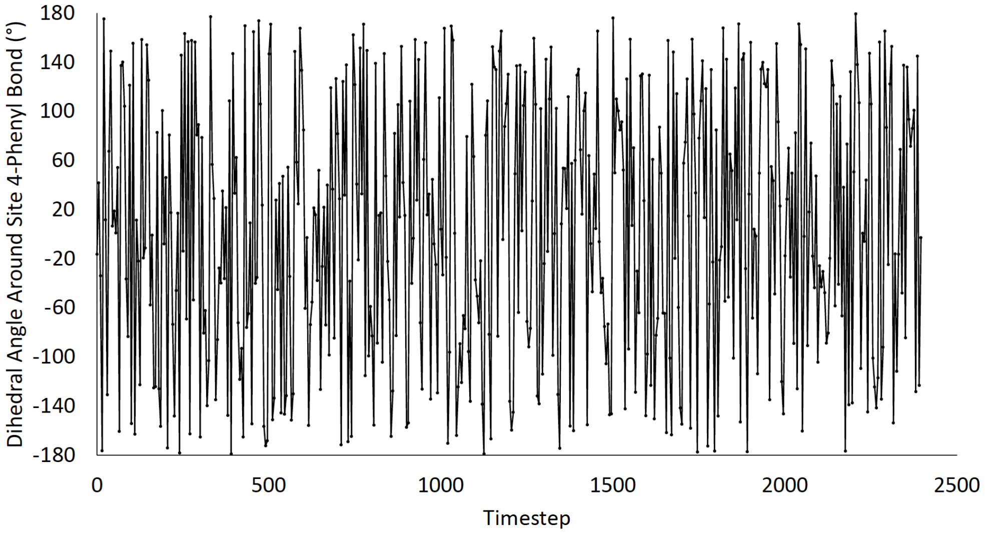

Figure S9: The change in dihedral angle of the site 4-phenyl torsion for the 3-pyridyl substituent (compound **4**) along the trajectory of the MSλD data collection stage.

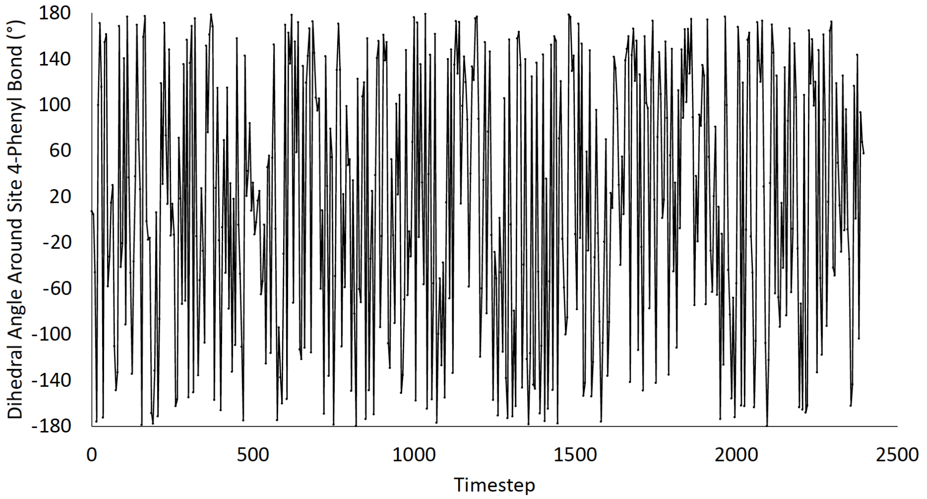

Figure S10: The change in dihedral angle of the site 4-phenyl torsion for the 1-ethylpyrazole substituent (compound **5**) along the trajectory of the MSλD data collection stage.

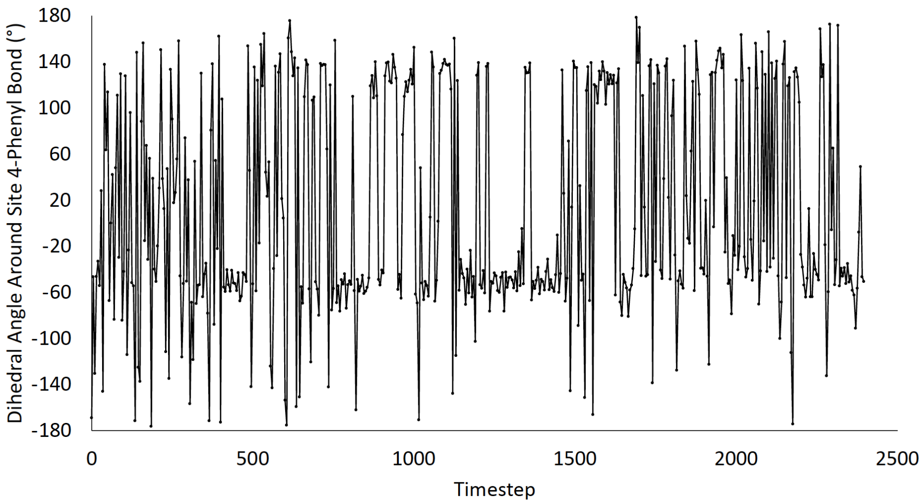

Figure S11: The change in dihedral angle of the site 4-phenyl torsion for the 3,5-dimethylisoxazole substituent (compound **7**) along the trajectory of the MSλD data collection stage.

References

(1) Wan, S.; Bhati, A. P.; Zasada, S. J.; Wall, I.; Green, D.; Bamborough, P.; Coveney, P. V. Rapid and Reliable Binding Affinity Prediction of Bromodomain Inhibitors: A Computational Study. *J. Chem. Theory Comput.* **2017**, *13*, 784–795.
