# Supplementary material for: Alchemical Free Energy Methods Applied to Complexes of the First Bromodomain of BRD4
Source: J Chem Inf Model. 2022 Mar 8;62(6):1458–70. doi: 10.1021/acs.jcim.1c01229 (PMC9098113; doi:10.1021/acs.jcim.1c01229)
Supplement: Supplementary file 3 — ci1c01229_si_003.pdf [file ci1c01229_si_003.pdf]

ID	SMILES
1	C[C@H](N1C(C)=O)C[C@@H](NC2=CC=CC=C2)C3=C1C=CC(C4=CC=C(OC)C=C4)=C3
2	C[C@H](N1C(C)=O)C[C@@H](NC2=CC=CC=C2)C3=C1C=CC=C3
3	C[C@H](N1C(C)=O)C[C@@H](NC2=CC=CC=C2)C3=C1C=CC(C4=CC=CC=C4)=C3
4	C[C@H](N1C(C)=O)C[C@@H](NC2=CC=CC=C2)C3=C1C=CC(C4=CN=CC=C4)=C3
5	C[C@H](N1C(C)=O)C[C@@H](NC2=CC=CC=C2)C3=C1C=CC(C4=CN(CC)N=C4)=C3
6	C[C@H](N1C(C)=O)C[C@@H](NC2=CC=CC=C2)C3=C1C=CC(C4=C(OC)C=CC=C4OC)=C3
7	C[C@H](N1C(C)=O)C[C@@H](NC2=CC=CC=C2)C3=C1C=CC(C4=C(C)ON=C4C)=C3
8	C[C@H](N1C(CC)=O)C[C@@H](NC2=CC=CC=C2)C3=C1C=CC(C4=CC=C(OC)C=C4)=C3
9	C[C@H](N1C(C(C)C)=O)C[C@@H](NC2=CC=CC=C2)C3=C1C=CC(C4=CC=C(OC)C=C4)=C3
10	C[C@H](N1C(C)=O)C[C@@H](NC2=CC=CC=C2)C3=C1C=CC(C4=CC=C(C[NH+]5CCCCC5)C=C4)=C3
11	CC(N1[C@@H](CC)C[C@@H](NC2=CC=CC=C2)C3=C1C=CC(C4=CC=C(C[NH+]5CCCCC5)C=C4)=C3)=O
12	CC(N1[C@@H](CCC)C[C@@H](NC2=CC=CC=C2)C3=C1C=CC(C4=CC=C(C[NH+]5CCCCC5)C=C4)=C3)=O
13	CC(N1[C@@H](CCC)C[C@@H](NC2=CC=CC=C2)C3=C1C=CC(C4=CC=C(C([O-])=O)C=C4)=C3)=O
14	CC(N1[C@@H](CC)C[C@@H](NC2=CC=CC=C2)C3=C1C=CC(C4=CC=C(C([O-])=O)C=C4)=C3)=O
15	C[C@H](N1C(C)=O)C[C@@H](NC2=CC=C(Cl)C=C2)C3=C1C=CC(C4=CC=C(C([O-])=O)C=C4)=C3
